# Molecular pathways, resistance mechanisms and targeted interventions in non-small-cell lung cancer

**DOI:** 10.1186/s43556-022-00107-x

**Published:** 2022-12-12

**Authors:** Zixi Wang, Yurou Xing, Bingjie Li, Xiaoyu Li, Bin Liu, Yongsheng Wang

**Affiliations:** 1 Thoracic Oncology Ward, Cancer Center, West China Hospital, Sichuan University, Chengdu, Sichuan China; 2 Clinical Trial Center, National Medical Products Administration Key Laboratory for Clinical Research and Evaluation of Innovative Drugs, West China Hospital, Sichuan University, Chengdu, Sichuan China; 3 State Key Laboratory Biotherapy, Cancer Center, West China Hospital, Sichuan University, Chengdu, Sichuan China; 4 Department of Medical Oncology, School of Medicine, Sichuan Cancer Hospital & Institute, Sichuan Cancer Center, University of Electronic Science and Technology of China, Chengdu, Sichuan China

**Keywords:** Non-small-cell lung cancer (NSCLC), Tyrosine kinase inhibitor, Resistance mechanisms, Targeted intervention

## Abstract

Lung cancer is the leading cause of cancer-related mortality worldwide. The discovery of tyrosine kinase inhibitors effectively targeting EGFR mutations in lung cancer patients in 2004 represented the beginning of the precision medicine era for this refractory disease. This great progress benefits from the identification of driver gene mutations, and after that, conventional and new technologies such as NGS further illustrated part of the complex molecular pathways of NSCLC. More targetable driver gene mutation identification in NSCLC patients greatly promoted the development of targeted therapy and provided great help for patient outcomes including significantly improved survival time and quality of life. Herein, we review the literature and ongoing clinical trials of NSCLC targeted therapy to address the molecular pathways and targeted intervention progress in NSCLC. In addition, the mutations in EGFR gene, ALK rearrangements, and KRAS mutations in the main sections, and the less common molecular alterations in MET, HER2, BRAF, ROS1, RET, and NTRK are discussed. The main resistance mechanisms of each targeted oncogene are highlighted to demonstrate the current dilemma of targeted therapy in NSCLC. Moreover, we discuss potential therapies to overcome the challenges of drug resistance. In this review, we manage to display the current landscape of targetable therapeutic patterns in NSCLC in this era of precision medicine.

**Supplementary Information:**

The online version contains supplementary material available at 10.1186/s43556-022-00107-x.

## Introduction

Lung cancer is the leading cause of cancer-related mortality in both males and females. The most common morphological subtype of lung cancer is non-small-cell lung cancer (NSCLC), which accounts for approximately 85% [1]. In 2004, a tyrosine kinase inhibitor (TKI) targeting epidermal growth factor receptor (EGFR) was reported to induce a dramatic tumor response compared with conventional chemotherapy in patients with NSCLC [2]. After that, the treatment landscape of this common cancer dramatically changed and experienced rapid growth. All great progress has been largely due to advances in tumor tissue analysis technologies such as next-generation sequencing (NGS) and the wider application of conventional methods such as immunohistochemistry (IHC), polymerase chain reaction (PCR), and fluorescence in situ hybridization (FISH) in the past several decades. In recent decades, the identification of actionable genetic alterations and the significantly improved patient outcomes of TKIs have altered the therapeutic algorithm for NSCLC patients [3]. Frontline EGFR-TKI therapy is the standard of care for NSCLC patients harboring EGFR mutations (exon 19 deletion or L858R mutation). Crizotinib also showed better efficacy than standard chemotherapy in advanced NSCLC patients with chromosomal rearrangements of the anaplastic lymphoma kinase gene (ALK) [4]. Benefitting from the tissue analysis technique, more targetable tumor mutations were identified, and TKIs were developed for clinical use from early-stage to advanced NSCLC. Generally, above the most reported mutation status, Kirsten rat sarcoma virus (KRAS), proto-oncogene receptor tyrosine kinase ROS1 (ROS1), V-raf murine sarcoma oncogene homolog B1 (BRAF), mesenchymal-epithelial transition factor (MET), human epidermal growth factor receptor (HER2), neurotrophic tyrosine kinase receptor (NTRK) chromosome rearrangements, and some other less established oncoproteins have also been reported and thoroughly investigated. With exploration of identified targetable mutations, the management of NSCLC has been transformed. Incidences of oncogenic driver alterations extracted from the studies showed that there were nearly 21.7 ~ 39.8% of patients with no actionable alteration. With respect of actional alterations of NSCLC, EGFR mutations account for approximately 18.9–51.4% of NSCLC, followed by KRAS mutations (11.2–32%), ALK rearrangements (3–7%), MET alterations (1–5%), HER2 alterations (3%), BRAF mutations (1.5–8%), ROS1 rearrangements (1–2%), RET mutations (1–2%), and NTRK mutations (less than 1%) [5–10]. The molecular alterations play more and more important roles in the management of NSCLC. According to the histological subtypes of NSCLC, lung adenocarcinoma (LUAD) and lung squamous carcinoma (LUSC) are associated with different mutational patterns, but are both mainly enriched for KRAS, EGFR, and ALK mutations [11].

Although the incidence of these oncogenic driver alterations is variable, their identification stimulated TKI exploration, and some of them significantly prolonged the overall survival (OS) of patients. While resistance inevitably occurs with the application of these TKIs, mechanistic analysis and further treatment strategies are under rapid investigation. Resistance mechanisms can be divided into primary and acquired. Primary resistance might be related to some intrinsic factors leading to unfavorable survival outcomes. Generally, acquired resistance can be divided into dependent and independent and arises from the acquisition of new mutations of the gene itself or in bypass or downstream pathways. Here, in this review, we will focus on the progress in understanding the molecular pathways, resistance mechanisms, and novel treatment strategies of NSCLC.

## EGFR activating mutations

### EGFR pathway and characteristics

EGFR, discovered in 1977 and coded on chromosome 7p11.2a, is a member of the ERBB receptor family [12]. It is a transmembrane protein with cytoplasmic kinase activity and an important signal transduction molecule that regulates cell proliferation and apoptosis [13]. After binding with a ligand, the conformation of EGFR changes, and dimers of the receptor form. Subsequently, autophosphorylation of amino acid residues occurs in the intracellular region of EGFR. Activated EGFR can transmit proliferation and anti-apoptotic signals to the nucleus through multiple downstream signal transduction pathways, such as PI3K/AKT/mTOR, RAS/RAF/MEK/ERK, and JAK/STAT, to control cell growth and division [14].

EGFR mutations are very frequent genetic alterations in NSCLC. EGFR mutations lead to the activation of downstream signaling pathways in the absence of ligand stimulation, which enhances metastasis and resistance to apoptosis, thereby promoting tumor development [15]. Statistically, EGFR mutations are significantly more frequent in patients who are females, nonsmokers, LUAD, and of Asian ethnicity [16]. Approximately 15% of Caucasian patients and 40–50% of Asian patients with LUAD  harbor EGFR gene mutations [17]. Most EGFR mutations occur in exons 18–21, with deletions in exon 19 (19 del) and L858R point mutations in exon 21 being the most common, and tumors harboring these mutations are sensitive to EGFR TKIs [18].

### EGFR TKIs

By binding with tyrosine kinase, EGFR TKIs can inhibit the activation of tyrosine kinase and block downstream signaling pathways, ultimately inhibiting the proliferation and metastasis of tumor cells and promoting the apoptosis of tumor cells. Before EGFR TKI’s advent, platinum-based chemotherapy had been the standard treatment for patients with advanced or metastatic NSCLC. In contrast with supportive care, chemotherapy only results in a marginal improvement in survival [19]. The development of targeted drugs has revolutionized the management of NSCLC, significantly prolonging OS and PFS in patients.

**Fig. 1:**
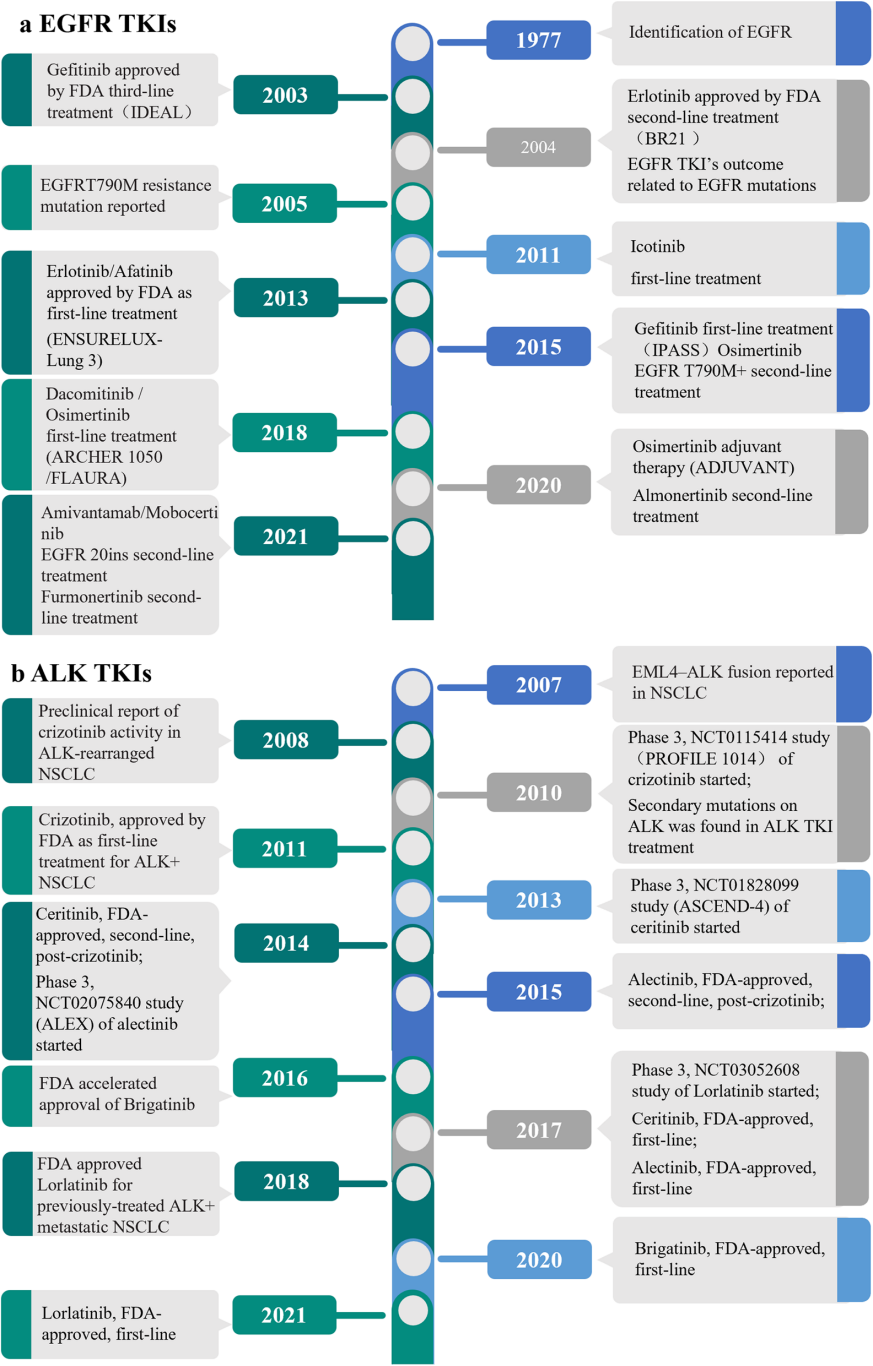
Development history in targeted therapy for NSCLC of EGFR TKIs and ALK TKIs. Timeline of breakthrough in EGFR-mutant NSCLC (part a) and ALK-positive NSCLC (part b). EGFR, epidermal growth factor receptor; ALK, anaplastic lymphoma kinase; NSCLC, non-small-cell lung cancer

At present, there are three generations of clinically available EGFR-TKIs (Fig. 1a): (1) the first generation of reversible inhibitors (gefitinib, erlotinib, and icotinib), (2) the second generation of irreversible inhibitors (afatinib, dacomitinib), and (3) the third generation of irreversible inhibitors (osimertinib, almonertinib, lazertinib, etc.) [20].

#### First- and second-generation EGFR-TKIs

A study in 2004 found that mutation of EGFR gene is correlated with clinical responsiveness to the TKI gefitinib in patients with NSCLC [21]. The IPASS study and the NEJ002 research showed that gefitinib prolongs median progression-free survival (mPFS) and enhances response rate compared with patients with advanced NSCLC with EGFR mutations who received chemotherapy [2, 22]. Subsequently, a large number of studies of gefitinib have been carried out worldwide. Given that gefitinib showed an advantage in improving efficacy and reducing toxicity for patients with NSCLC, it was recommended as a first-line treatment for NSCLC patients with sensitive mutations in the EGFR gene in July 2015. EURTAC and CONVINCE research supported erlotinib and icotinib, respectively, as standard first-line therapies in advanced NSCLC due to improved PFS and ORR [23, 24]. Follow-up comparative studies illustrated that there was no significant difference among the three first-generation drugs in efficacy and toxicity, and they all did not achieve significant OS benefits compared with chemotherapy [25].

Despite the high disease control rates, drug resistance inevitably emerges within approximately 10 months after treatment and leads to disease progression. Therefore, it is necessary to develop a new generation of drugs that can delay or overcome acquired resistance. The second-generation EGFR TKI afatinib is an irreversible inhibitor of the EGFR tyrosine kinase. Two studies investigated the efficacy of afatinib compared with chemotherapy in an EGFR-mutant population, the LUX-Lung 3 and LUX-Lung 6 studies. The results showed that mPFS was improved in the afatinib group [26–28]. In particular, OS was improved in patients with lung adenocarcinoma harboring the EGFR 19 del mutation but not in patients with EGFR L858R or in the EGFR mutation-positive patient population overall [29]. In addition, afatinib has shown a better treatment response than gefitinib, bringing longer remission in patients [30]. In 2013, afatinib was approved by the US Food and Drug Administration (FDA) for first-line treatment for NSCLC patients with EGFR mutation. Above the potential treatment efficacy in patients with 19 del and L858R, the results of the combined analysis of LUX‐lung 2, 3, and 6 trials supported afatinib as the first choice in certain types of rare EGFR mutations, especially Gly719Xaa, Leu861Gln, and Ser768Ile, demonstrating clinical benefit in patients [31]. In the ARCHER 1050 study, another second-generation EGFR-TKI, dacomitinib, provided PFS and OS benefits over gefitinib in the first-line treatment of NSCLC patients with EGFR mutations [32]. In September 2018, the FDA approved dacomitinib as a first-line treatment for advanced or metastatic NSCLC patients with EGFR mutations, providing more options for these patients. Dacomitinib also shows superiority in OS, especially in the subgroup with exon 21 L858R substitution mutation [33]. The response difference of 19 del and L858R to afatinib and dacomitinib indicated that the biological difference may exist between 19 del and L858R mutations. It was reported that patients with 19 del were found to have longer survival than those with the L858R mutation. A possible explanation might be that the L858R mutation is accompanied by a more frequent appearance of EGFR exon 20 Thr790Met (T790M) mutations and concomitant mutations [34]. Disappointingly, second-generation TKIs fail to solve the problem of drug resistance, which remains a serious challenge for the treatment of NSCLC.

#### Third-generation EGFR-TKIs

The mechanisms of drug resistance are quite complex. More than half of the resistance to first- and second-generation EGFR TKIs is due to a new mutation called T790M, which was first identified in 2005. This mutation results in a steric hindrance effect that weakens the binding ability of EGFR TKIs and increases the affinity of the EGFR mutant to ATP, resulting in acquired resistance to TKIs [35]. To overcome this critical mutation in EGFR-TKI resistance, novel generation TKIs are urgently needed to solve this problem and benefit patient survival. Osimertinib, the first third-generation EGFR TKI, inhibits both EGFR classical mutations and T790M mutations and demonstrated better efficacy in advanced NSCLC patients with T790M-positive compared with chemotherapy. The median duration of PFS was significantly longer with osimertinib than with chemotherapy [36]. The FDA accelerated the approval of osimertinib for the treatment of NSCLC patients with EGFR T790M mutation in November 2015. Based on the promising results of a series of clinical trials, in April 2018, the FDA further approved osimertinib as a first-line treatment for metastatic NSCLC patients with EGFR mutations (19 del or 21 L858R). The FLAURA clinical trial demonstrated that osimertinib extended PFS by 8.7 months compared with first-generation TKIs and showed efficacy in patients with NSCLC who had central nervous system (CNS) metastases [37]. In addition, the randomized ADAURA trial also showed a significant benefit from receiving osimertinib as adjuvant therapy after resection of stage IB-IIIA lung cancer with typical EGFR mutations. The results of this study indicated that osimertinib reduces the risk of disease recurrence or death by 83% and prevents the occurrence of postoperative brain metastases [38, 39]. On December 19, 2020, the FDA officially declared osimertinib as the first adjuvant therapy for patients with NSCLC with EGFR mutations.

Almonertinib is China's first self-developed third-generation EGFR-TKI. In the APOLLO study, almonertinib improved PFS and had a significant effect on EGFR mutation-positive NSCLC patients with brain metastases [40, 41]. Based on these outcomes, in March 2020, almonertinib was conditionally approved by the National Medical Products Administration (NMPA) for second-line treatment of T790M positive patients. In December 2021, it was officially approved as the first-line treatment for patients with locally advanced or metastatic NSCLC with EGFR 19 del or L858R mutations based on AENEAS trail.

Many other third-generation targeted drugs, such as furmonertinib and lazertinib, have also shown good antitumor effects in NSCLC patients with the T790M mutation [42]. The LASER201 study showed that with lazertinib as a second-line treatment for patients with the EGFR T790M mutation, the ORR and PFS were 57.9% and 11.0 months, respectively [43]. In addition, lazertinib appeared an encouraging intracranial activity and clinical studies on the first-line treatment of lazertinib are currently underway. On 18 January 2021, lazertinib was approved for the treatment of EGFR T790M mutation-positive NSCLC after progression on prior EGFR TKIs. Lazertinib in combination with amivantamab increases efficacy in patients with osimertinib resistance. The ORR was 36%, and the clinical benefit rate was 60% [44]. The phase III MARIPOSA study for Lazertinib plus amivantamab versus osimertinib as first-line therapy in EGFRm NSCLC is ongoing (NCT04487080). Nazartinib (EGF816) is another third-generation EGFR TKI that blocks both T790M and EGFR classical mutations. In patients with EGFR mutations who failed prior to therapy, the ORR was 51%, and PFS was 9.1 months. The ORR among those with EGFR T790M and 19 del mutations was higher (61%) than that among those with EGFR T790M and L858R mutations (35%) [45]. A series of clinical trials on third-generation targeted drugs are ongoing (Table 1).

**Table 1:** Clinical trials of EGFR TKIs

Experimental drug	Target	Development stage	Condition	Status	Primary endpoint	Study results	NCT number
Third generation EGFR-TKIs							
AZD3759	EGFR	Phase II/III	EGFR + NSCLC with BM	Active, not recruiting	PFS	Not available	NCT03653546
Rezivertinib (BPI-7711)		Phase III	Advanced treatment-naïve EGFR + NSCLC	Active, not recruiting	PFS	Not available	NCT03866499
D-0316		Phase II/III	Locally advanced or metastatic EGFR + NSCLC	Active, not recruiting	PFS	Not available	NCT04206072
Abivertinib (AC0010)		Phase III	Advanced EGFR + NSCLC	Not yet recruiting	PFS	Not available	NCT03856697
ZN-e4		Phase I	Advanced EGFR + NSCLC	Active, not recruiting	DLTs	Not available	NCT03446417
ASK120067		Phase III	Locally advanced or metastatic EGFR + NSCLC	Recruiting	PFS	Not available	NCT04143607
XZP-5809-TT1		Phase I	Locally advanced or metastatic T790M + NSCLC	Recruiting	AEs, ORR, PFS, blood routine	Not available	NCT04622072
Oritinib(SH-1028)		Phase III	Locally advanced or metastatic T790M + NSCLC	Not yet recruiting	PFS	Not available	NCT04239833
CK-101		Phase I/II	EGFR + NSCLC	Active, not recruiting	DLTs, ORR	Not available	NCT02926768
Lazertinib (YH25448)		Phase III	Locally advanced or metastatic EGFR + NSCLC	Active, Not recruiting	PFS	Not available	NCT04248829
BPI-15086		Phase I	EGFR T790M + NSCLC	Completed	AEs	Not available	NCT02914990
TY-9591		Phase III	Advanced EGFR + NSCLC	Not recruiting	PFS	Not available	NCT05382728
Fourth generation EGFR-TKIs							
FWD1509	EGFR	Phase I/II	EGFR + NSCLC	Recruiting	AEs	Not available	NCT05068024
TQB3804	EGFR Del19/T790M/ C797S EGFR L858R/ T790M/C797S EGFR Del19/T790M EGFR L858R/T790M	Phase I	EGFR + NSCLC	Unknown	DLTs	Not available	NCT04128085
BPI-361175	EGFR C797S	Phase I/II	Locally advanced or recurrent/metastatic EGFR + NSCLC	Recruiting	AEs, RP2D, ORR	Not available	NCT05329298
BLU-701	EGFR L858R/ C797S EGFR Del19/ C797S	Phase I/II	EGFR + NSCLC	Recruiting	MTD, RP2D, ORR, AEs	Not available	NCT05153408
BLU-945	EGFR L858R/T790M/ C797S	Phase I/II	EGFR + NSCLC	Recruiting	PR2D, ORR, AEs	Not available	NCT04862780
BBT-176	EGFR C797S	Phase I/II	EGFR + NSCLC	Recruiting	DLTs, ORR	Not available	NCT04820023
Tagerting EGFR 20ins							
Poziotinib	EGFR 20ins	Phase II	EGFR or HER2 20 ins NSCLC	Recruiting	ORR	Not available	NCT03318939
PLB1004		Phase I	Advanced NSCLC EGFR + or HER2 +	Recruiting	AEs, DLTs, MTD, RP2D	Not available	NCT05347628
BLU-451		Phase I/II	EGFR 20 ins advanced Lung Cancers	Recruiting	MTD, DLTs, RP2D, AEs, ORR	Not available	NCT05241873
DZD9008		Phase II	EGFR or HER2 + NSCLC	Recruiting	MTD, PR2D, ORR	Not available	NCT03974022
TAK-788		Phase III	NSCLC EGFR 20 ins	Recruiting	PFS	Not available	NCT04129502
CLN-081		Phase I/IIa	NSCLC EGFR 20 ins	Recruiting	AEs, DLTs, ORR	Not available	NCT04036682
Multi-target drugs							
FCN-411	pan-HER	Phase I/II	Advanced NSCLC EGFR +	Recruiting	Cmax, AUC, Tmax, t1/2	ORR = 14.9%, DCR = 73.1%, mPFS = 4.1 m	NCT03420079
Keynatinib	EGFR/BTK	Phase II	NSCLC EGFR + With BM	Recruiting	ORR	Not available	NCT04824079
Pyrotinib	EGFR/ HER2	Phase II	NSCLC EGFR + or ERBB2 20 ins	Unknown	ORR	Not available	NCT04063462
Tesevatinib	Multi-target	Phase II	EGFR + NSCLC	Completed	ORR	Not available	NCT02616393
EGFR antibody							
MRG003	EGFR	PhaseII	EGFR-Positive Advanced Non-Small Cell Lung Cancer	Recruiting	ORR	Not available	NCT04838548
M1231	MUC1/EGFR	Phase I	mNSCLC experssing EGFR and Mucin 1	Recruiting	DLTs, AEs,OR,DoR	Not available	NCT04695847
EMB-01	EGFR/MET	PhaseI/II	Advanced/metastatic NSCLC EGFR + and/or c-MET +	Recruiting	MTD, AEs,ORR	Not available	NCT03797391
MCLA-129	EGFR/MET	Phase I/II	Advanced NSCLC, EGFR + and/or MET +	Recruiting	DLTs, MTD, ORR, AEs	Not available	NCT04930432
HLX35	Anti-EGFR/Anti-4-1BB	Phase I	EGFR + advanced Squamous NSCLC	Not yet recruiting	AEs, DLTs, MTD, RP2D	Not available	NCT05360381
BCA101	EGFR/TGFβ	Phase I	EGFR + Squamous Cell Carcinoma of the Lung	Recruiting	DLTs, AEs	Not available	NCT04429542
U3-1402 (patritumab deruxtecan)	HER3	Phase III	EGFR + metastatic or unresectable NSCLC	Recruiting	PFS	Not available	NCT05338970

DLTs, Dose limiting toxicities; RP2D,Recommended Phase 2 Dose; ORR,Overall Response Rate; MTD, Maximum tolerated dose; PFS, Progression-free Survival; AEs: Adverse events; DoR, Duration of response; t1/2, elimination half life; Tmax, time of maximum blood concentration; Cmax, maximum observed blood drug concentration; AUC, area under the blood concentration–time curve

### Treatment of uncommon EGFR mutations

Except for the typical mutations, approximately 2% of NSCLC patients harboring EGFR exon 20 insertions, which is the third most common type of EGFR mutation [18]. Patients harboring EGFR exon 20 insertions have lower response rates with EGFR TKIs and worse prognoses than those sensitizing EGFR mutations [46, 47]. High-dose osimertinib (160 mg daily) was tried as a treatment in advanced-stage NSCLC patients with EGFR exon 20 insertions, which showed limited clinical activity [48]. Efforts to target this rare but difficult-to-treat mutation continue, and the recently published antitumor efficacy of amivantamab brings hope to this group of patients [49]. Amivantamab (JNJ-61186372) is a fully human EGFR-MET bispecific antibody. By binding to EGFR and the extracellular domain of the c-MET receptor, it blocks the binding of ligand and receptor, thus inhibiting the activation of downstream signaling. In addition, it can induce the degradation of receptors and NK-dependent antibody-dependent cell-mediated cytotoxicity (ADCC) to eliminate antigen-expressing tumor cells [46, 50]. In the CHRYSALIS trial, patients with EGFR exon 20 insertion mutations after progression on platinum-based chemotherapy responded well to amivantamab (ORR = 40%) [47]. Another agent, mobocertinib, is a novel oral EGFR/HER2 dual-targeted drug. The EXCLAIM study reported that the ORR was 25% in NSCLC patients with EGFR 20 insertions after progression with chemotherapy [51]. Mobocertinib provides a choice in EGFR 20 insertions-positive mNSCLC [48]. A novel TKI CLN-081 causes persistent tumor regression in EGFR exon 20ins-driven mouse models [20]. Early Phase I studies reported a confirmed ORR of 31% [52]. Data from the ongoing WK-KONG1 and WU-KONG2 studies suggested that sunvozertinib is active in NSCLC pre-treated patients with EGFR Exon20ins, and the ORR is 37.5% [53]. The above results bring hope for patients harboring EGFR exon 20 insertions.

However, effective therapies have not been identified for some other uncommon EGFR mutations, including 18 del, Glu709Xaa, and 19 ins. The effects of atypical EGFR mutations on drug sensitivity are unknown. Recent studies have found that the previous method of predicting drug sensitivity based on exon location classification is unscientific and that classification based on structural changes in EGFR mutations can more effectively guide the treatment of EGFR-mutated NSCLC patients. The structural-based four-category classification could more effectively personalize EGFR TKI therapy and drug development [54]. More research is needed to further benefit patient survival.

### Resistance mechanisms and treatment strategy of EGFR TKIs

**Fig. 2:**
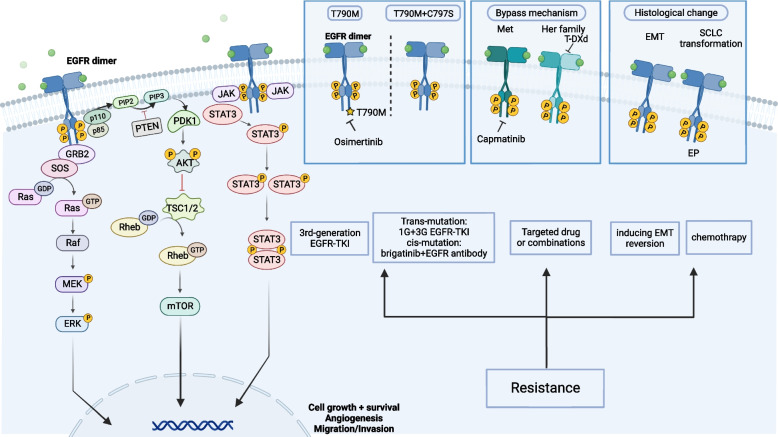
Signaling pathway, resistance mechanisms, and treatment strategies of EGFR. Overview of the EGFR signaling pathway, with a focus on major resistance mechanisms and related potential treatment strategies. After binding with the ligands, EGFR forms a dimer, thereby activating downstream signaling pathways, such as PI3K/AKT/mTOR, RAS/RAF/MEK/ERK, and JAK/STAT, to control cell growth and division (as shown on the left). The acquired resistance mechanisms to EGFRtargeted therapies include secondary mutation of EGFR gene, activation of alternative or downstream signaling pathways, and tumor histological transformation. Secondary mutation of EGFR gene includes EGFR T790M, C797S, and others. MET and HER2 are identified as bypass pathway resistance alterations. With regard to histological transformation, it was reported cases related with EMT and histological transformation to SCLC. Some of the latest treatments against EGFR TKIs resistance are also briefly shown in the figure(as shown on the right). EGFR, epidermal growth factor receptor; PI3K, phosphatidylinositide 3-kinases; mTOR, mechanistic target of rapamycin; JAK, Janus kinase; STAT, signal transducer and activator of transcription; MET, hepatocyte growth factor receptor; HER2, human epidermal growth factor receptor; EMT, epithelial to mesenchymal transition; SCLC, small cell lung cancer

Above T790M, the mechanisms of resistance to EGFR TKIs are varied, including MET amplification, KRAS mutation, BRAF mutation, PIK3CA mutation, SCLC transformation, PTEN deletion, etc. [55]. Generally, there are four main types of mechanisms involved. (Details were presented in Fig. 2).

#### EGFR-dependent resistance

EGFR C797S mutation is one of the most common mechanisms against osimertinib. In the EGFR C797S mutation, osimertinib cannot covalently bind with the mutant EGFR. There are two types of mutations in EGFR C797S: trans-mutations and cis-mutations. Transmutations are sensitive to the combination of first-generation and third-generation TKIs. However, patients with cis-mutations show resistance to all available EGFR TKIs [20]. In some studies, brigatinib combined with an anti-EGFR antibody proved efficacy in the treatment of EGFR C797S cis-mutations [56]. A study showed that the fourth-generation of EGFR TKIs, EAI045 in combination with cetuximab is effective in mouse models of lung cancer with EGFR L858R/T790M/C797S mutations. Other fourth-generation EGFR-targeted agents, including BLU-701 (NCT05153408) and BLU-945 (NCT04862780), are recently being found effective in treating triple-mutant EGFR. There are also a number of fourth-generation drugs being investigated (Table 1). There are several preclinical drugs that have been shown to inhibit the growth of triple-mutated tumor cells, producing sustained antitumor effects, such as JBJ-04–125-02, BI-4020, JND3229, CH7233163, AZ7608. These drugs are currently in active development and are expected to enter clinical trials soon [20, 57–60]. Other rare EGFR tertiary mutations including L792X, G796S, L718Q, and G724S mutations cause drug resistance by affecting the binding of osimertinib to EGFR [61].

#### Activating mutations of bypass pathways resistant to EGFR TKIs

Amplification of the MET oncogene is observed in approximately 20% of resistance cases. By binding to the ligand hepatocyte growth factor (HGF), it activates the PI3K/AKT/mTOR signaling pathway and leads to drug resistance [62]. In an EGFR TKI-resistant cell line with acquired MET amplification, the response to EGFR TKI treatment could be restored by using the MET inhibitor [63]. Regimens targeting both EGFR and MET amplification appear to be the mainstream treatment option for this resistance. Capmatinib plus gefitinib and the combination of osimertinib and savolitinib improved the ORR in patients with EGFR-mutated, MET-dysregulated NSCLC [64, 65]. Similarly, in patients with MET overexpression or MET amplification, the PFS and OS are longer by using the combination of tepotinib plus gefitinib compared with chemotherapy [66]. In the CHRYSALIS trial cohort, amivantamab and lazertinib also improved the ORR in osimertinib-resistant patients, especially in EGFR- and MET-positive patients. The above findings showed that the combination of MET inhibitors with EGFR inhibitors is a promising choice for drug resistance treatment strategies. HER2 amplification is another potential mechanism of resistance to EGFR TKIs in NSCLC. Trastuzumab (a monoclonal antibody against HER2) and Drug conjugates of trastuzumab may provide a novel therapeutic approach for lung cancers with HER-2 amplification [67, 68]. Furthermore, HER3-DXd (an ADCs targeting HER3) was found to be effective in EGFR-mutated drug-resistant non-small cell lung cancer [69]. In addition, acquired resistance induced by ALK or RET rearrangement can occur in some patients, which can be overcome by the combination of EGFR TKI and ALK or RET inhibitors [70, 71].

Activation of the insulin-like growth factor 1 receptor (IGF1R) signaling pathway contributes to EGFR-TKI resistance in NSCLC patients, and the IGF1R pathway might be a promising target for overcoming resistance [72]. AXL is a member of the TAM family of receptor tyrosine kinases (RTKs). On the basis of recent data, the up-regulation of AXL is one of the mechanisms of acquired resistance to TKIs in EGFR-mutated NSCLC. Aberrant expression of AXL promotes epithelial-mesenchymal transition (EMT), and activates MAPK, PI3K/AKT and NF-κB signals to enhance tumor cell survival and metastasis. AXL-induced drug resistance was reversed by treatment with inhibition of AXL signaling [73].

#### Activating mutations of downstream pathways resistant to EGFR TKIs

Recent studies suggest that the activation of RAS/MAPK, PI3K/AKT, or STAT3 signaling independent of EGFR could be a frequent resistance mechanism to TKIs [74]. KRAS inhibitors have the potential to overcome drug resistance induced by KRAS mutation [75]. The use of EGFR TKI and a MEK inhibitor can delay or prevent resistance to EGFR TKIs in EGFR mutation and NRAS/BRAF mutation tumors [76, 77]. PTEN loss is correlated with the activation of PI3K/mTOR signaling and shows a poor prognosis. Thus, using targeted inhibitors (PI3K/mTOR/Akt) to block PTEN loss-driven signaling pathways might produce antioncogenic effects [78]. Lapatinib (a dual TKI of EGFR and HER2) or afatinib (a pan-TKI of EGFR family proteins) can effectively inhibit the activation of PI3K/AKT. It can restore the sensitivity of EGFR TKI treatment [79]. Similarly, PX-866, a Ptdins-3-kinase inhibitor, potentiates the antitumor activity of gefitinib [80]. The mTOR inhibitor and JAK2 inhibition improve EGFR TKI outcomes [81, 82]. Findings from preclinical studies showed proto-oncogene tyrosine-protein kinase (Src) SRC activation can mediate the acquisition of erlotinib resistance, and the SRC inhibitor dasatinib effectively inhibited EGFR TKI-resistant cell survival [83, 84]. It suggests that the combination of EGFR-TKI and other signaling pathway inhibitors is a promising treatment strategy.

#### Histological transformation resistant to EGFR TKIs

A recent study shows that histological changes might be correlated with the acquisition of EGFR TKI resistance. The process of the loss of epithelial phenotypes and the gain of mesenchymal features is called EMT, which has been reported to be associated with increased capacity for migration and invasion of tumor cells [85]. Recent advances have suggested that TGF-β induces EMT by activating the Smad3, PI3K/AKT/mTOR, and MEK/ERK cascades [86]. PP242 (a mTOR inhibitor) and metformin have been reported to restore gefitinib-induced apoptosis by inducing EMT reversion and have the potential to overcome drug resistance in cancer cells [87]. Small cell lung cancer (SCLC) transformation is an important and infrequent resistance mechanism. The exact mechanisms leading to this transformation are not well understood. Previous studies have shown that NSCLC patients with TP53, RB1, and EGFR genetic alterations are more likely to develop small cell transformation. Transformed SCLC is generally sensitive to chemotherapy [85, 88]. In a very small number of patients, the tumor type changes from adenocarcinoma to squamous carcinoma [89]. This change of phenotype indicated a generally poor prognosis.

#### Other mechanisms resistant to EGFR TKIs

Besides the mechanisms discussed above, some other mechanisms are also involved in EGFR TKI resistance. Recently, Niu et al. reported that FBXL2 can induce the degradation of EGFR and EGFR TKI-resistant mutations. Reduced FBXL2 expression is associated with poor clinical outcomes in NSCLC patients. FBXL2 activation in combination with EGFR-TKIs or Grp94-specific inhibitors is a kind of strategy that needs further investigation in EGFR-resistant NSCLC [90]. NF-κB is a transcription factor that regulates cell proliferation, apoptosis, and inflammation, and its activation has been reported to be correlated with resistance to several EGFR TKIs. The direct NF-κB inhibitor PBS-1086 increased the duration of the initial EGFR inhibitor response in multiple NSCLC mouse models [91]. Knocking down FAS and several components of the NF-κB signaling pathway would explicitly enhance cell death which was induced by erlotinib in EGFR-mutant lung cancer cells [92]. BCL2L11 (also known as BIM) encodes a BH3-only protein that activates cell death, and its upregulation is required for TKIs to induce apoptosis. The prevalence of BIM deletion polymorphism in East Asian individuals is 12.3%. The change affects the function of promoting apoptosis and impairs the clinical efficacy of targeted drugs, predicting a significantly shorter PFS [93]. APG-1252, as a dual inhibitor of Bcl-2/Bcl-xL, has shown a synergistic antitumor effect with EGFR-TKIs in EGFR-mutated NSCLC. P16/CDKN2A inactivation can affect the regulation of cell cycle and is correlated with primary resistance to EGFR-TKIs in NSCLC patients [94]. However, none of the drugs targeting these mechanisms have thus far been applied in clinical settings. The efficacy of targeted therapy alone is unsatisfactory; hence, a variety of combination therapies are needed.

### Combination therapy with EGFR TKIs

#### Targeted therapy combined with antiangiogenic therapy

Angiogenesis is a key step in the development and metastasis of solid tumors [95]. Targeting vascular endothelial growth factor (VEGF) has shown improved survival in many cancers. Dual inhibition of EGFR and the VEGF pathway is an effective anticancer strategy in advanced NSCLC. The ARTEMIS study demonstrated a significant improvement in PFS of 6.7 months after adding the VEGF inhibitor bevacizumab to the EGFR inhibitor erlotinib [96, 97]. Additionally, comparing ramucirumab plus erlotinib with the placebo plus erlotinib, targeted therapy combined with antiangiogenic therapy showed prolongation of PFS (19.4 months vs 12.4 months) [98]. The CTONG1706 study investigated the efficacy and safety of apatinib and gefitinib combination therapy, showing a longer mPFS in patients treated with apatinib and gefitinib compared with placebo and gefitinib (13.7 months vs. 10.2 months) [99]. However, based on available data, osimertinib does not appear to be appropriate for combination therapy in patients with advanced NSCLC. PFS was not longer in the osimertinib plus bevacizumab group than in the osimertinib group in patients with advanced lung adenocarcinoma with EGFR T790M mutation [100]. Taken together, EGFR-TKIs combined with antiangiogenic therapy are a new feasible treatment strategy for the treatment of EGFR-mutated metastatic NSCLC.

#### Targeted therapy combined with chemotherapy

Updated analyses have confirmed that PFS was improved with a combination of chemotherapy and targeted therapy in advanced NSCLC patients with EGFR mutation. The FASTACT-2 study showed that six cycles of gemcitabine plus platinum and erlotinib achieved better therapeutic effects than chemotherapy alone [101]. The NEJ009 study also demonstrated that gefitinib combined with carboplatin plus pemetrexed showed a prolongation of PFS in advanced NSCLC patients with EGFR-mutation compared with gefitinib alone. The side effects of the combination therapy did not increase significantly [102]. The combination of chemotherapy and targeted therapy is a viable first-line option for patients with EGFR mutations.

#### Targeted therapy combined with immunotherapy

Retrospective analysis demonstrated that the frequency of PD-L1 expression and response rates to anti-PD-1/PD-L1 antibody among EGFR-mutant patients was relatively low [103]. However, some other studies reported that EGFR-TKI treatment can increase the expression of PD-L1 [104]. In the TATTON study, there was no obvious benefit of combining durvalumab with osimertinib in comparison with osimertinib monotherapy. Combination therapy was associated with a higher incidence of adverse events, such as interstitial lung disease (ILD) or ALT/AST elevation [65, 105]. The phase III clinical trial CAUREL demonstrated that nivolumab in combination with EGFR-TKI increased the risk of interstitial pneumonitis compared with either drug alone [106]. The literature reported that ipilimumab plus erlotinib and gefitinib plus tremelimumab caused excessive gastrointestinal toxicity in advanced NSCLC patients with EGFR mutation, preventing further evaluation of this combination [107, 108]. Another study found that nivolumab plus erlotinib was tolerated and showed clinical activity in NSCLC patients with EGFR mutation previously treated with TKIs [109]. The discordance of these studies may be attributed to different reasons, such as the sample size of the study, previous treatment history, tumor mutation burden, and PD-L1 expression. In summary, the clinical application of the combination of EGFR TKI and immunotherapy needs further investigation.

#### Other combination strategies with EGFR TKIs

Studies confirmed that afatinib plus cetuximab, a monoantibody targeting EGFR, is not superior to afatinib alone in the treatment of NSCLC patients. There was no improvement in PFS in patients receiving combination treatment, and the toxicity was greater in the combination group [110]. In addition, some case reports revealed that patients clinically benefited from combination therapy of cabozantinib (a multi-target small molecule TKI) and osimertinib after osimertinib resistance [111]. The IMpower150 trial showed improved PFS and OS in atezolizumab plus bevacizumab and chemotherapy versus the standard-of-care bevacizumab plus chemotherapy in patients with non-squamous NSCLC [112]. With the development of novel targets and treatments in cancer, more combination strategies may arise to benefit patients after critically designed studies. Some combination strategies have also entered the clinical stage of testing, as presented in Supplementary Table 1.

## KRAS G12C mutations

Kirsten rat sarcoma (KRAS) is the most frequent isoform in the RAS family, the most common oncogene family in human cancer [113], and KRAS accounts for approximately 85% of mutations in RAS-mutant cancer [114]. KRAS is a membrane-bound guanosine triphosphatase (GTPase) that acts as a molecular switch, which changes between active and inactive by binding to guanosine triphosphate (GTP) or guanosine diphosphate (GDP) [114, 115]. The majority of KRAS mutations in NSCLC occur in codons 12 or 13, and the most frequent mutation is a transversion in which amino acid glycine is replaced by cysteine (G12C variants), accounting for 39–41% of KRAS mutations, followed by G12V (19–21%) and G12D (14–17%) [116, 117]. KRAS mutations account for up to 32% of patients with NSCLC [118, 119]. Dysregulation of KRAS resulting from point mutations can directly affect downstream signaling pathways, especially the PI3K/Akt and MAPK pathways [120], thus leading to abnormal tumor growth [121].

### KRAS G12C-targeted therapies

Since KRAS mutations were discovered, great efforts have been made to develop targeted therapies, including directly targeting KRAS and indirectly targeting downstream signaling pathways, posttranslational modifications, protein-protein interactions, and membrane localization [122]. However, no specific targeted strategies have been reported for a long time, and KRAS was acknowledged as “undruggable” due to the absence of drug-binding pockets on the surface of RAS proteins [123]. Remarkably, sotorasib (AMG510), a RAS GTPase family inhibitor, was approved by the US FDA in May 2021 and was the first approved targeted treatment for KRAS G12C-mutated NSCLC [124]. Sotorasib demonstrated durable clinical benefits to pretreated KRAS G12C-positive patients without new safety signals and superior efficacy to previous standard chemotherapy. A randomized open-label, phase I/II, CodeBreak 100 study was conducted in 733 patients with KRAS G12C mutation advanced solid tumors and in a subgroup of 126 NSCLC patients pretreated with standard therapies. There were 46 patients observed an objective response (ORR = 37.1%), and the mPFS was 6.8 months [125]. A clinical trial CodeBreak 200 is ongoing to compare sotorasib (AMG510) with docetaxel in pretreated NSCLC patients with KRAS G12C mutation. (NCT04303780).

Currently, desirable approaches to treating KRAS-mutant tumors consist of directly and indirectly inhibiting KRAS. Specific KRAS inhibitors mainly include sotorasib and adagrasib (MRTX849), which were designed on the basis of the switch-II pocket and target KRAS G12C irreversibly [126]. Adagrasib is a small molecular KRAS inhibitor that can distribute into tissue widely to cross the blood-brain barrier, helping to maximize drug effectiveness with a 24-h half-life period [115]. With a recommended dose of 600 mg, the mPFS was 11.1 months in 25 patients with advanced KRAS G12C solid tumors in the KRYSTAL-1 trial [127]. Recently, a multicenter phase III trial (KRYSTAL-12, NCT04685135) designed to compare the efficiency and safety of adagrasib versus docetaxel is ongoing in 452 previously treated NSCLC patients. Similarly inhibiting KRAS G12C directly, GDC-6036 and D1553 also showed promising clinical activity in phase I clinical trials [128, 129]. Besides, a variety of additional drugs are also being developed to inhibit KRAS indirectly. These include inhibition of SHP2, RMC-4630, a selective, potent, and orally bioavailable allosteric inhibitor of SHP2. A phase I, multicenter study of oral RMC-4630 monotherapy in patients with relapsed/refractory solid tumors is underway (NCT03634982). Another phase I trial of JAB-3068 in adult patients with advanced solid tumors is also ongoing (NCT03518554). Promising results have also been expected for the SOS1 inhibitor BI-1701963. Currently, a phase I trial of BI-1701963 monotherapy and combination therapy with trametinib to test different doses in KRAS-mutant patients is ongoing (NCT04111458).

### Resistance mechanisms to KRAS G12C-targeted therapies

Clinical efficiency has been observed in KRAS-targeted therapies; however, primary or acquired resistance to monotherapies still occurs in patients [130–131]. Awad et al. performed genomic and histologic analyses among patients with KRAS G12C-positive cancers treated with adagrasib, which conferred acquired resistance mechanisms to KRAS G12C inhibitors [132]. The acquired resistance mechanisms mainly include secondary KRAS mutations and KRAS amplification, new KRAS, activating mutations of bypass pathways, and histological transformation [133].

#### Primary resistance mechanisms to KRAS TKIs

**Fig. 3:**
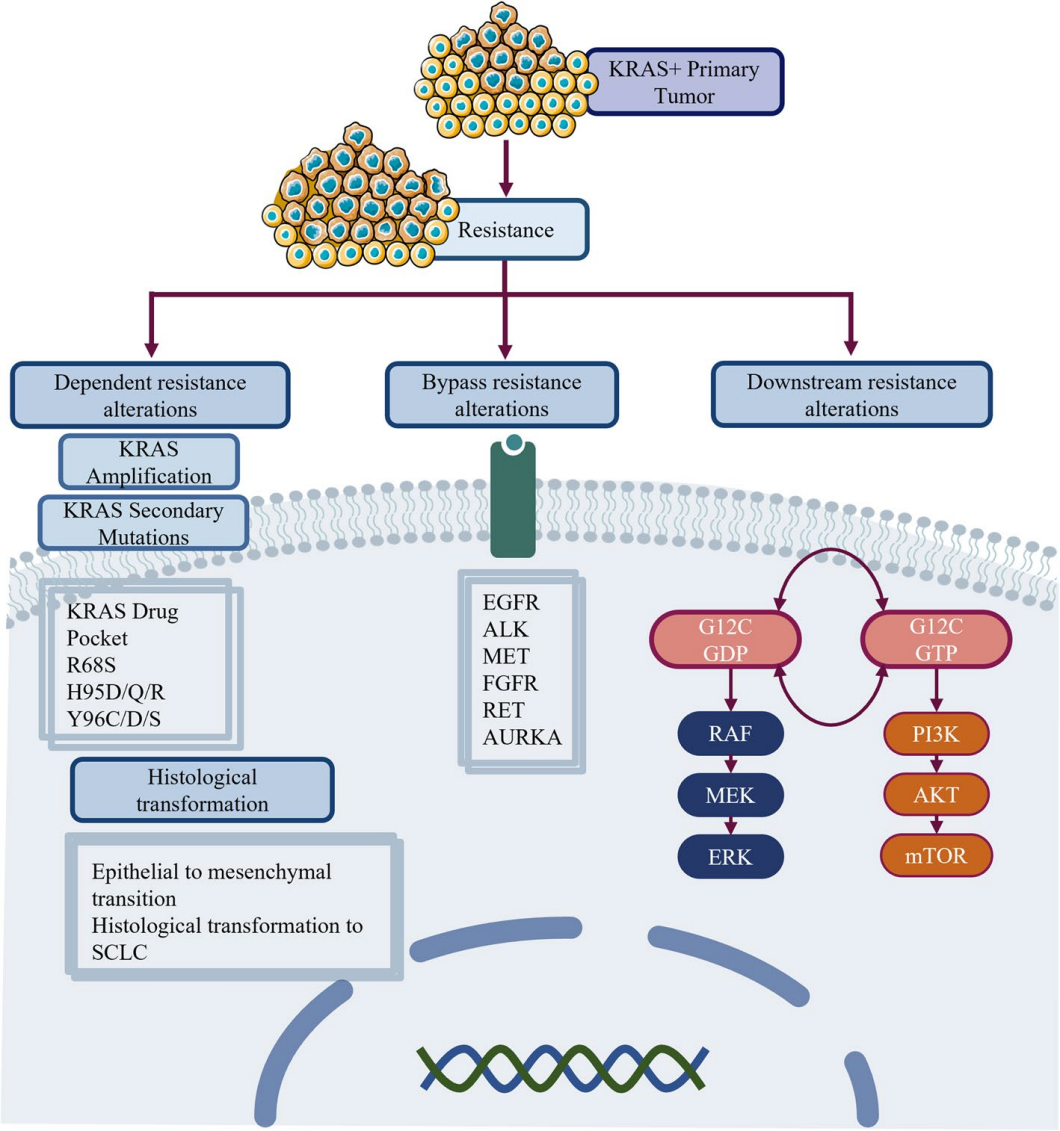
Resistance mechanisms of KRAS TKIs. Acquired resistance mechanisms to KRAS targeted therapies in NSCLC can be mainly divided into KRAS-dependent and KRAS-independent resistance mechanisms. KRAS-dependent resistance mechanisms include KRAS amplification and KRAS secondary mutations. KRAS-independent resistance mechanisms include bypass resistance alterations, downstream resistance alterations, and histological transformation. EGFR, ALK, MET, FGFR, RET, and AURKA are identified as bypass pathway resistance alterations. In addition, mutations of BRAF, MEK, mTOR, and JAK are identified as downstream pathway resistance alterations. With regard to histological transformation, it was reported cases related with EMT, and histological transformation to SCLC. KRAS, Kirsten rat sarcoma virus; TKIs, tyrosine kinase inhibitors; EGFR, epidermal growth factor receptor; ALK, anaplastic lymphoma kinase; MET, hepatocyte growth factor receptor; FGFR, fibroblast growth factor receptor; RET, proto-oncogene tyrosine-protein kinase receptor Ret; BRAF, V-raf murine sarcoma oncogene homolog B1; mTOR, mechanistic target of rapamycin; JAK, Janus kinase; EMT, epithelial to mesenchymal transition; SCLC, small cell lung cancer

According to the CodeBreak 100 study, the ORR of sotorasib is lower than that of TKIs targeting other driver mutations, which might be due to the inherent molecular heterogeneity of KRAS-mutant tumors [125]. This heterogeneous response which is potentially caused by low dependency on the KRAS signaling pathway appears to be attributable to the primary or intrinsic resistance mechanisms. Tumor growth is mainly associated with two downstream pathways (MAPK and PI3K), in which activation of PI3K is mediated by not only KRAS but also other upstream signals independent of KRAS. Singh et al. found that KRAS dependency varied widely in KRAS-mutant tumors, and they established a “Ras Dependency Index” (RDI) to evaluate KRAS dependency [134]. In some KRAS-independent cell lines, even when KRAS was completely suppressed, the cells could still survive, suggesting that the resistance of some KRAS G12C mutated tumors to G12C inhibitors might be due to the low dependency on KRAS [135]. In addition, EMT has been confirmed as a cause of both intrinsic and acquired resistance mechanisms, which activate the PI3K pathway and are predominantly regulated by the IGFR-IRS1 pathway [136]. The resistance mechanisms of KRAS have been presented in Fig. 3.

#### Secondary KRAS mutations and KRAS amplification

Drug-binding site mutations (KRAS Y96C, R68S, and H95D/Q/R mutations) within the switch II pocket binding to adagrasib and sotorasib were described. Y96C and R68S conferred resistance to sotorasib, Y96C, R68S, and H95D/Q/R conferred resistance to adagrasib. The analysis of crystallographic structure showed that these mutations could lead to disruption of noncovalent binding interactions of the inhibitors and stronger interactions of R68 and Y96 with sotorasib. In contrast, H95D/Q/R remained sensitive to sotorasib [132]. Furthermore, Y96D/S mutations were also identified as secondary resistant mutations to inhibitors in vitro which were sensitive to the combination therapy of SOS1 inhibitor, BI-3406, and trametinib [137]. According to the structural modeling, the Y96D mutation disrupts vital hydrogen bonding between the carboxyl group on sotorasib and Y96, as well as the pyrimidine ring of adagrasib and the hydroxyl group of Y96. Additionally, the Y96D mutation switches the hydrophobic pocket to be more hydrophilic, which may weaken the stability of binding [138]. Deep mutational scanning positively selected the resistant mutations. Codons 8, 9, 64, 99, and 117 and multiple mutations in codons 12, 68, 95, and 96 conferred strong resistance to adagrasib. Codons 8, 9, 12, 96, and 117 mutations were observed in the sotorasib screen. In addition, mutations at codons 13, 59, 61, 117, and 146 promoting GDP to GTP nucleotide exchange or hindering GTP hydrolysis were detected and described to be associated with resistance to both drugs [132]. KRAS G12C allele amplification at a high level was observed in two adagrasib-resistant samples without other resistance mechanisms, while in another study, low-level KRAS copy number gain in three patients treated with sotorasib was detected [132, 139].

#### New KRAS G12C

KRAS G12C inhibitors solely inhibit the inactive conformation of KRAS G12C, and the subpopulations of isogenic tumor cells respond nonuniformly to the inhibitors [140]. Xue et al. found that only those cells in the GDP conformation could be strongly inhibited and become quiescent or undergo apoptosis, while others respond insensitively to the inhibitors and could mediate reactivation of the MAPK pathway [140]. Integrating the results from differential analysis and genome-wide knockout-screen, it was found that heparin-binding epidermal growth factor (HBEGF) activates EGFR to induce KRAS activation and enhance signaling in an EGFR/SHP2-dependent manner. Furthermore, aurora kinase A (AURKA) interacts with KRAS to facilitate effector c-Raf activation and cell cycle progression. Based on the results of vitro and vivo models, J.W. Lee et al. confirmed antitumor activity for AURKA inhibition with sotorasib in lung cancer with intrinsic resistance to KRAS G12C inhibitors [141]. The results indicated that HBEGF and AURKA could mediate cells with the new KRAS G12C to escape from inhibitors and resume proliferation.

#### Activating mutation of bypass pathways resistant to KRAS TKIs

Adaptive feedback reactivation has hindered previous clinical attempts targeting the RAS-MAPK pathway. Some preclinical findings have suggested that signaling adaptation plays a potential role in limiting the efficacy of the KRAS G12C inhibitors such as ARS-1620 [142]. RTKs are the second largest family of membrane receptors, which can transfer extracellular signals to the intracellular domain and mediate a series of downstream effects, such as cell growth, migration, and metastasis [143]. A variety of RTKs, including EGFR, are important upstream factors in the activation of KRAS. Upstream RTK regulators (EGFR, FGFR, HER2, c-MET, and SHP2), direct mediators of KRAS activation (AURKA), and/or MYC and mTOR may tissue-specifically escape from inhibition [144]. In the study of KRAS G12C cell lines treated with AMG-510 and ARS-1620, it was observed that RAS pathway feedback signaling was driven by multiple RTK-mediated activations of wild-type RAS. It was also found that SHP2 mediated signaling from RTK to RAS, which suggested SHP2 and KRAS G12C inhibitors combined therapy could improve efficacy in vitro and in vivo [145]. Indeed, SHP2 mediates multiple RTK signals to the RAS pathway, and SHP2 inhibition inhibits RTK-mediated feedback signals in various tumor models in vitro, xenografts, and syngeneic KRAS G12C*-*mutant pancreatic ductal adenocarcinoma (PDAC) and NSCLC. The combination of SHP2 and KRAS G12C inhibition induced favorable but tumor site-specific changes in the immune microenvironment, increasing CD8 + T cells, decreasing myeloid suppressor cells, and sensitizing tumors to PD-1 blockade [146]. In preclinical models acquiring resistance to the KRAS G12C inhibitors, activated MET maintained the active form of RAS mediated by SOS1, subsequently activating the MAPK pathway, which suggests that MET amplification causes resistance to KRAS G12C inhibitors [147].

#### Histological transformation resistant to KRAS TKIs

Histological transformation, including transformation from adenocarcinoma to squamous cell carcinoma and EMT, has been identified, similar to other targeted therapies observed in NSCLC. Awad et al. observed histological transformation from adenocarcinoma to squamous cell carcinoma in two NSCLC patients, in whom deep targeted panel sequencing and ctDNA sequencing were performed [132]. EMT was also identified as one of the acquired resistance mechanisms of sotorasib in KRAS G12C-mutant NSCLC lines. Adachi et al. found that the IGFR-dependent KRAS G12C-independent activation of PI3K-AKT signaling in EMT-induced KRAS G12C-mutant cancer cells. The upregulated IGFR signal appeared to dominantly regulate PI3K–AKT activation. Furthermore, FGFR was also involved in the activation of EMT-related resistance to molecular targeted therapy [136].

### Combination therapy with KRAS TKIs

Despite these preliminary encouraging results in clinical trials of inhibitors targeting KRAS mutations, patients are unlikely to benefit enduringly from monotherapy [148]. Considering the poor benefit of monotherapy and possible resistance to inhibitors, combination therapies are still essential. One of the combination strategies is direct inhibitors in combination with indirect inhibitors of KRAS. Several preclinical studies demonstrated that adagrasib in combination with SHP2 inhibitors led to tumor regression in several KRAS G12C mutant patient-derived and cell line xenograft models [115]. At present, several clinical trials to evaluate the efficacy and safety of the combination of a KRAS G12C inhibitor, sotorasib or adagrasib, and an SHP2 inhibitor are ongoing (NCT05054725, NCT04330664). And it has been observed promising clinical activity and well tolerance in the combination treatment of sotorasib and RMC-4630 (an SHP2 Inhibitor) [149]. Combination therapy of KRAS G12C inhibitor adagrasib with the SOS1 inhibitor BI-1701963 is also being studied recently (NCT04975256). Potential strategies also focus on combining KRAS inhibitors with inhibition of the upstream EGFR pathway and downstream pathways, including MAPK and PI3K. Several preclinical trials have tested sotorasib in combination with inhibitors of EGFR, SHP2, MEK, PI3K, and AKT. It was found that combination with MEK inhibitors had the greatest synergy in vivo [130]. Similar preclinical studies were also conducted in adagrasib combined with inhibitors of ErbB, MEK, and mTOR, and they all showed superior efficacy to monotherapies [115]. However, the treatment efficacy of combined therapy needs further investigation and randomized clinical trials. Whether the combination will increase the toxicity also needs to be addressed.

Several studies have claimed the superior efficacy of immune checkpoint inhibitors (ICIs) in KRAS-mutant NSCLC [150, 151]. The possible mechanism of the superior response to ICIs is that KRAS mutation is related to tumor immunogenicity and the inflammatory tumor microenvironment [152]. As the long-term efficacy of sotorasib in the KRAS G12C mutation model depends on the engagement of the immune system, and ICIs such as anti-PD-L1 may synergize with sotorasib [130]. Besides, it was found that MAPK pathway activation might contribute to immune evasion and lead to poor recurrence-free survival [153]. A phase Ib/II nonrandomized study (CodeBreak 101) is ongoing to explore the efficiency of sotorasib in combination with PD-1 and PD-L1 inhibitors (NCT04185883). Recently, the study reported that the combination of sotorasib and immunotherapy increased hepatotoxicity, and the efficacy needed further confirmation [154].

## ALK gene rearrangements

Echinoderm microtubule-associated protein-like 4 (EML4) and ALK fusion within chromosome 2p were first discovered in NSCLC by Soda and his colleagues in 2007 [155]. The ALK gene encodes a member of the insulin receptor superfamily, which is a highly conserved transmembrane receptor tyrosine kinase involved an intracellular domain, an extracellular domain, and a transmembrane region [156, 157]. The EML4-ALK fusion gene leads to overactive expression and activation of ALK with the consequence of upregulation of cell proliferation and survival [158, 159]. In addition, EML4-ALK fusion proteins interact with a complicated network of bypass and downstream pathways, such as EGFR, KIT, JAK/STAT3, MEK/ERK, and PI3K/AKT [157, 160]. The EML4-ALK fusion transcript occurs in 3 ~ 7% of all NSCLC cases, and these individuals were distinct from those harboring mutations in the EGFR gene [155, 161–163]. After the EML4-ALK fusion transcript was first discovered, inhibitors targeting this mutation have started and experienced rapid development, exemplified by the global approval of ALK TKIs such as first-generation inhibitors (crizotinib), second-generation inhibitors (ceritinib, alectinib, and brigatinib), and third-generation inhibitors (lorlatinib). The development history in targeted therapy for NSCLC of ALK TKIs is shown in Fig. 1b.

### ALK TKIs

#### First-generation ALK TKIs

The first-generation ALK TKI crizotinib had substantial benefits with respect to ORR, PFS, and quality of life compared to standard chemotherapy [164, 165]. The FDA approved crizotinib firstly for the treatment of patients with metastatic or locally advanced ALK-positive NSCLC on August 26, 2011, based on the results of two clinical trials, including a phase I study (PROFILE 1001, NCT00585195) and a phase II study (PROFILE 1005, NCT00932451), which filled the gap of targeted therapies in the field of ALK-positive NSCLC at that time [166]. The confirmation of clinical benefit was based on PROFILE 1014, and the mPFS was 10.9 months in the crizotinib group versus 7.0 months in the chemotherapy group [165]. However, CNS progression and the appearance of new intracranial lesions were common during crizotinib treatment. A retrospective study involving PROFILE 1005 and PROFILE 1007, patients with unpretreated brain metastases (BM) had an intracranial DCR of 56% and an intracranial time to progression (TTP) of 7 months; patients pretreated for BM had an intracranial DCR of 62% and a TTP of 13.2 months [167]. The possible mechanism was that poor accumulation and penetration of crizotinib in the CNS resulted in a predisposition toward CNS metastases during crizotinib treatment [168, 169].

#### Second-generation ALK TKIs

Within a year or two after crizotinib treatment, drug resistance and CNS metastases inevitably emerge. Except for the mechanisms of CNS metastases mentioned above, the resistance mechanisms include on- and off-target resistance, and second-generation ALK TKIs have therefore been developed.

Ceritinib is an ATP-competitive and selective second-generation ALK TKI that shows 20-fold higher selectivity over crizotinib [170]. Ceritinib inhibits not only ALK but also secondary mutations of ALK, including L1196M, G1269A, S1206Y, and I1171T EML4-ALK mutants [171], and IGF1R, which plays a significant role in tumor growth and is overexpressed in several CNS metastases [170, 172, 173]. According to the results of the ASCEND-4 study, ceritinib had superior improvement in mPFS compared with chemotherapy (16.6 *vs.* 8.1 months) [174]. In the phase III ASCEND-5 trial, patients who progressed on crizotinib and chemotherapy were recruited in this study, and the study demonstrated that ceritinib improved PFS versus chemotherapy (5.4 *vs.* 1.6 months) [175]. To enhance GI tolerability, a phase I ASCEND-8 study was conducted and demonstrated that 450 mg ceritinib with food was associated with lower GI toxicities [176].

Alectinib prevents autophosphorylation of ALK and suppresses phosphorylation of STAT3 and AKT and shows activity against the gatekeeper L1196M mutant [177]. The final PFS analysis of ALEX showed that alectinib has the longest mPFS of 34.8 months in the first-line setting, providing 3 times longer efficacy benefit compared with crizotinib (10.9 months) [178]. In addition, 12% (18/152) of patients in the alectinib group had CNS progression, as it was 45% (68/151) in the crizotinib group [179]. Alectinib shows protective and preventive effects on brain metastases, and the possible mechanism is that alectinib could not efflux from the CNS through the P-glycoprotein-mediated transporter in a study of intracranial tumor models of ALK-positive NSCLC [179, 180].

Another second-generation ALK TKI, brigatinib, shows 12-fold greater potency than crizotinib and maintains substantial antitumor activity against 17 secondary ALK mutants [181]. The final results of the ALTA-1L study demonstrated that the 3-year PFS by BIRC was 43% and 19%, respectively (the mPFS was 24.0 *vs.* 11.1 months) [182]. A meta-analysis showed that the efficacy of alectinib ranked the highest in the whole patient population, while in the CNS metastatic patient subgroup, brigatinib ranked the highest by efficacy [183]. The results may be explained by the chemical structure of brigatinib, which has a dimethylphosphine oxide (DMPO) group, which alectinib does not have, and the structure may lead to high affinity for NSCLC cells and low lipophilicity, low protein-binding capacity, and high water solubility, which contributes to the activity of brigatinib in the CNS [181, 184]. And brigatinib showed meaningful clinical efficacy in patients progressed in alectinib in ALTA-2 or J-ALTA clinical trials [185].

#### Third-generation ALK TKIs

The third-generation ALK TKI lorlatinib is highly selective and inhibits ALK and ROS1 tyrosine kinases [186], which has been shown to be effective against almost of known resistant mutants, including the G1202R mutant [187]. Its higher CNS permeability compared with previous generations of ALK TKIs has been confirmed [188], and a PET imaging scan showed that after intravenous injection of [^11^C] lorlatinib in rhesus macaque models, high initial uptake was observed in the brain, and it was highest concentrated in the cerebellum, frontal cortex, and thalamus [189]. A randomized, multicenter, phase III trial compared lorlatinib with crizotinib. The rate of patients without disease progression in 12 months was remarkably higher in the lorlatinib group (78%) than in the crizotinib group (39%), and the ORRs were 76% and 58%, respectively [190].

### Resistance mechanisms to ALK inhibitors

**Fig. 4:**
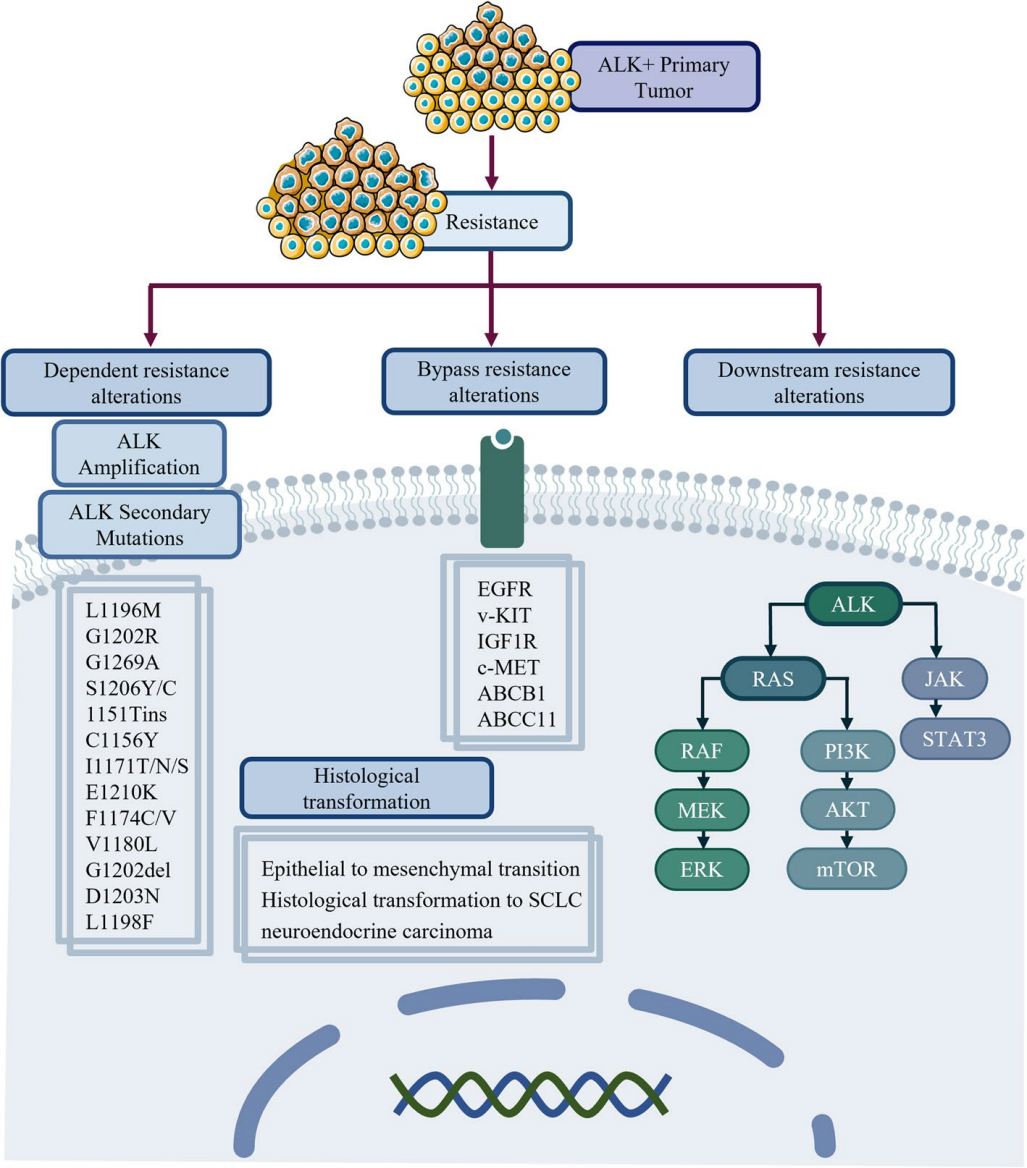
Resistance mechanisms of ALK. Acquired resistance mechanisms to ALK TKIs in NSCLC can be mainly divided into ALK-dependent and ALK-independent resistance mechanisms. ALK-dependent resistance mechanisms include ALK amplification and ALK secondary mutations. ALK-independent resistance mechanisms include bypass resistance alterations, downstream resistance alterations and histological transformation. EGFR, v-KIT, IGF1R, c-MET, ABCB1, and ABCC11 are identified as bypass pathway resistance alterations. In addition, mutations of BRAF, KRAS, MEK, mTOR, and JAK are identified as downstream pathway resistance alterations. With regard to histological transformation, it was reported cases related with EMT, histological transformation to SCLC, and neuroendocrine carcinoma. ALK, anaplastic lymphoma kinase; TKIs, tyrosine kinase inhibitors; EGFR, epidermal growth factor receptor; v-KIT, Hardy-Zuckerman 4 feline sarcoma viral oncogene homolog; IGF1R, insulin-like growth factor 1 receptor; MET, hepatocyte growth factor receptor; ABCB1, ATP binding cassette subfamily B member 1; ABCC11, ATP-binding cassette subfamily C member 11; BRAF, V-raf murine sarcoma oncogene homolog B1; KRAS, Kirsten rat sarcoma virus; mTOR, mechanistic target of rapamycin; JAK, Janus kinase; EMT, epithelial to mesenchymal transition; SCLC, small cell lung cancer

Although ALK TKIs have achieved good efficacy in patients with ALK-positive NSCLC, most patients will inevitably eventually develop drug resistance and metastases to other sites, such as the brain or liver. ALK-positive patients can have disease progression because of various resistance mechanisms with a median period from 10.9 months to 34.8 months [191]. According to the timeline of occurrence, resistance mechanisms can be classified into "de novo" or "acquired" [192, 193]. Apart from a small number of patients with primary resistance to ALK TKIs, most of the patients have acquired drug resistance. The mechanism of acquired drug resistance is mainly classified into ALK-dependent resistance and ALK-independent resistance. The resistance mechanisms of ALK have been presented in Fig. 4.

#### Primary resistance to ALK TKIs

It was reported that 6.5% (28/428) of patients with crizotinib treatment was observed primary resistance who displayed unfavorable survival outcomes, and the mPFS of the primary-resistance responder group was 1.2 months, while it was 47.0 months in the long-term responder group [194]. Since there are few studies on primary resistance to ALK TKIs, the potential mechanism of drug resistance is not yet clear. Several case reports revealed possible intrinsic factors of primary resistance to crizotinib, including MYC amplification [195], ALK/KRAS co-alteration [196, 197], Bim deletion polymorphism [198], high tumor mutational burden (TMB), and mutations in DNA repair genes (including TP53 G245S) [199]. More efforts should be made to explore the mechanisms of primary resistance to improve the treatment efficiency of these patients.

#### ALK-dependent resistance

ALK-dependent resistance can be divided into ALK fusion gene amplification and ALK kinase domain mutations, in which ALK secondary mutations were observed in 20% of crizotinib treatment and in 56% of second-generation TKIs treatment, and ALK amplification accounted for approximately 8% [200, 201].

ALK kinase domain mutations have various mutation locations, including gatekeeper mutation, solvent front, covalent binding site, and compound mutations [192, 202]. The possible mechanisms of secondary mutations are that kinase domain mutations affect the binding of drugs to active sites by increasing steric hindrance, activating the conformation of ALK, and permanently phosphorylating ALK [203]. Drug resistance mutation sites of crizotinib include L1196M, G1202R, G1269A, S1206Y/C, 1151Tins, C1156Y, I1171T/N/S, and E1210K. L1196M gatekeeper mutation and G1296A are the most common mutations in crizotinib treatment and hinder crizotinib binding to the ATP-binding pocket [157]. Second-generation ALK TKIs overcome most resistance to crizotinib, including L1196M and G1269A; however, the G1202R mutation has become the most common resistance in patients with second-generation ALK TKIs, while it is rare in crizotinib treatment [204]. There are some differences among the second-generation ALK TKIs. Ceritinib resistance mutations mainly include F1174C/V, G1202R, and G1202del, and alectinib resistance mutations mainly include I1171T/N/S, G1202R, and V1180L. Brigatinib has been demonstrated to have activity against all 17 crizotinib-, ceritinib- and alectinib-resistant secondary ALK mutants in a preclinical model and to demonstrate antitumor activity against the G1202R mutant [181]. However, the G1202R mutation was still observed in patients treated with brigatinib in a retrospective study [205]. Other resistance mutations, including E1210K, D1203N, and S1206Y/C, were also identified in brigatinib-resistant specimens [184]. Lorlatinib has been reported to be sensitive against resistance mutations of first- and second-generation ALK TKIs, including the highly refractory G1202R solvent front mutation [204]. Despite the remarkable efficacy of lorlatinib, acquired resistance persists and causes disease progression, and L1198F is the first drug-resistant mutation of lorlatinib to be identified in 2016 [206]. Subsequent reports suggested that compound ALK mutations were the main secondary mechanism of resistance to loratinib (such as C1156Y/L1198F, I1171N/D1203N, G1202R/L1196M, and others), which interfered sterically with drug binding or reduced potency of lorlatinib (not achieved the required clinical concentration) [206, 207]. The resistance mutations could gradually accumulate during treatment to culminate in highly refractory compound mutations challenging to be targeted [208, 209]. Another important reason for tumor progression is ALK fusion gene amplification, which results in the failure of crizotinib to completely inhibit downstream signaling [210, 211].

#### ALK-independent resistance

ALK-independent resistance includes activation of bypass or downstream pathways [192] and histological transformations (such as small cell transformation and neuroendocrine transformation) [212–215].

The activation of bypass or downstream signaling pathways induces resistance against ALK TKIs via EGFR, v-kit, IGF1R, MAPK, c-MET, and yes-associated protein (YAP) signaling pathways [216–221]. IGF1R was upregulated in ALK TKI-resistant cells, and when stimulating cells pretreated with crizotinib with IGF1, ALK phosphorylation was inhibited. Ceritinib can inhibit ALK and IGF1R, which is effective in crizotinib-resistant ALK-positive NSCLC [172]. Resistance caused by MET activation usually could not be observed in crizotinib treatment because crizotinib is an ALK and MET TKI. However, MET amplification has significantly increased in second-generation TKI treatment, which provided the reason for the feasibility of combination therapy of ALK and MET TKIs [222]. The RAS/MAPK pathway has been identified as one of the vital downstream pathways of ALK, and upfront inhibition of ALK and MEK could be a strategy to postpone resistance and improve outcomes [223]. YAP is one of the major downstream effectors of the Hippo pathway regulating tumorigenesis [224]. YAP1 is associated with the initial survival of ALK-rearranged cells against alectinib by regulating pro-apoptotic proteins [221].

Besides, other alternative signaling pathways include changes in tumor histology (EMT) and abnormal protein expression (overexpression of p-glycoprotein) [225]. The histological transformation to SCLC has been reported in crizotinib-resistant cases [226]. In addition, in a case of acquired resistance to lorlatinib, transformation to a neuroendocrine carcinoma was observed [214]. EMT was associated with a higher expression of zinc finger E-box binding homeobox 1 (ZEB1) and a lower expression of miR-200c, which led to cross-resistance to ceritinib, alectinib, and lorlatinib [227]. Crizotinib and ceritinib exhibited poor activity in CNS progression induced by overexpression of p-glycoprotein (ATP binding cassette subfamily B member 1, ABCB1), and it seemed that alectinib and lorlatinib could overcome the resistance [225]. Even so, it has recently been reported that overexpression of ATP-binding cassette subfamily C member 11 (ABCC11) may represent a mechanism involved in resistance to alectinib in a preclinical model [228].

### Novel ALK inhibitors

Initial success of all-generation ALK inhibitors has been observed in ALK-positive NSCLC patients; however, these ALK inhibitors have limitations due to various drug resistance mechanisms. Increasing attention has been given to resistance mechanisms, and new drugs targeting these mechanisms are constantly being developed. Solving these underlying resistance mechanisms is a clinical challenge, and it is critical to investigate novel treatments to overcome previous ALK TKIs acquired drug resistance. The current TKIs in clinical trials against ALK-rearranged NSCLC are listed in Table 2.

**Table 2:** Current novel ALK TKIs in clinical trials

Drug	Development stage	Condition	Status	Primary endpoint	Study results	NCT number
Repotrectinib (TPX-0005)	Phase I/II	Patients with locally advanced or metastatic solid tumors harboring ALK, ROS-1, or NTRK1-3 rearrangements	Recruiting	Phase I: DLT, RP2D; Phase II: ORR	Not available	NCT03093116
TPX-0131	Phase I/II	Patients with ALK + advanced or metastatic NSCLC pretreated	Recruiting	Phase I: DLT, RP2D; Phase II: ORR	Not available	NCT04849273
Ensartinib(X-396)	Phase I/II	Patients with advanced solid tumors; patients with advanced ALK-positive NSCLC	Completed	MTD	ALK-positive patients: RR = 60%; mPFS = 9.2 months	NCT01625234
					ALK- naïve patients: RR = 80%; mPFS = 9.0 months	
WX-0593	Phase III	Patients with ALK-positive NSCLC who had not received prior systemic therapy	Recruiting	PFS	Not available	NCT04632758
Alkotinib Capsules	Phase II	Patients with ALK-positive NSCLC previously treated with crizotinib	Recruiting	ORR	Not available	NCT04211922
TQ-B3139	Phase III	Patients with ALK-positive NSCLC that have received one chemotherapy regimen and have not received ALK inhibitor	Recruiting	PFS	Not available	NCT04009317
CT-707	Phase I	Patients with ALK-positive NSCLC	Recruiting	DLT, MTD/RP2D	Not available	NCT02695550
PLB1003	Phase I	Patients with advanced ALK-positive NSCLC	Recruiting	DLT, MTD	Not available	NCT03130881
XZP-3621	Phase I	Patients with ALK or ROS1-positive NSCLC	Recruiting	AEs, DLT, MTD/RP2D	Not available	NCT05055232
SY-3505 (CT-3505)	Phase I	Patients with ALK-positive NSCLC	Recruiting	AEs, DLT, MTD/RP3D	Not available	NCT05257512
SAF-189 s	Phase I/II	Patients with ALK or ROS1-positive NSCLC	Recruiting	Phase I: DLT; Phase II: ORR	Not available	NCT04237805

DLT, Dose limiting toxicities; RP2D,Recommended Phase 2 Dose; ORR,Overall Response Rate; MTD, Maximum tolerated dose; PFS, Progression-free Survival; AE: Adverse Event

### Combination therapies with ALK TKIs

To inhibit the emergence and existence of resistant tumor cells and improve the efficiency of treatment, potential strategies are to develop comprehensive treatment, including combination with bypass resistance inhibition, immunotherapy, or radiotherapy [229]. Although there is still no established treatment combined with ALK TKIs at present, many efforts to explore available strategies have been made [230]. Strategies with an ALK TKI combined with other agents targeting bypass pathways, such as MEK, may be a promising approach [229]. Hrustanovic et al. demonstrated that EML4-ALK lung adenocarcinoma depends innately on RAS-MAPK signaling via mechanisms including DUSP6 downregulation or KRAS amplification [223]. Shrestha et al. combined crizotinib with selumetinib, a MEK inhibitor, to investigate the effects on crizotinib-resistant ALK-positive lung cancer cells and found that the combination therapy could reduce the survival of tumor cells more evidently than single therapies [231]. These studies demonstrated that ALK TKIs combined with MEK TKIs targeting ALK-positive lung cancer cells have superior efficiency at suppressing cell growth, which provides an alternative approach for treating ALK-positive patients. Another combination therapy can be ALK TKIs combined with a MET kinase inhibitor. Crizotinib, the first-generation ALK TKI, was originally developed to inhibit MET and ROS1. With low blood-brain barrier penetration capability, crizotinib can be used in the treatment of MET- and ALK-positive patients without CNS metastases [222]. However, acquired resistance to crizotinib associated with MET exon 14 skipping has been reported [232]. Thus, combining a MET TKI with another ALK TKI, such as ceritinib, is a rational strategy. Otherwise, the combination of chemotherapy, especially pemetrexed-based regimens or chemoimmunotherapy with high CNS penetration targeted therapy, may be effective in patients with brain metastases or systemic oligoprogression [229, 233]. With respect to the combination of immunotherapy with ALK TKIs, the underlying benefits are still unclear. Few data on immune checkpoint monotherapy for ALK-positive patients indicates reduced efficacy [234]. Several early phase trials combining ALK TKIs with immunotherapy are ongoing: atezolizumab in combination with alectinib (NCT02013219), ipilimumab or nivolumab in combination with crizotinib (NCT01998126).

## Uncommon mutations of NSCLC

Since EGFR activating mutations were identified and EGFR TKIs were explored, the personalized medicine landscape of NSCLC has been leading to a dramatic revolution. Except the most reported EGFR, KRAS, and ALK mutations, a variety of low prevalence (less than 5%) oncogenic drivers including MET, HER2, BRAF, ROS1, RET, and NTRK, have been identified over the last decade with new technologies such as NGS and PCR [235]. Compared to the mature development of those mutations, targeted therapeutic treatment of those novel targeted drivers has far less developed, but those mutations are getting more and more attention.

### MET alterations

MET gene is a proto-oncogene located at 7q21–q31 [236]. The MET gene encodes a receptor tyrosine kinase (RTK) transmembrane protein called cellular mesenchymal epithelial transition factor (c-MET), also named hepatocyte growth factor receptor (HGFR) [237, 238]. The HGF/c-MET axis normally plays an important role in cell proliferation, differentiation, and migration. After binding with HGF, c-MET activation stimulates downstream signaling pathways via different membrane receptors, such as MAPK and PI3K [237]. The dysregulation of the HGF/c-MET axis is closely related to malignant cellular transformation, invasion, and metastasis [239]. Abnormal MET pathway activation is characterized by MET exon 14-skipping mutations (METex14), MET overexpression, and MET amplification [240]. MET overexpression can be related to many factors, including the hypoxic environment and transcriptional upregulation of the receptor [241]. It is the most frequent in MET pathway activation; however, its incidence can vary widely in different studies, ranging from 15 to 70% [242]. It has been proven that MET amplification is a primary oncogenic driver gene as well as one of the critical mechanisms of EGFR TKIs resistance. The original MET amplification was reported approximately 1–5% in NSCLC [243]. METex14 occurs in about 3% to 4% of NSCLC cases, and it is more likely for NSCLC patients with METex14 to have a smoking history [244].

#### Strategies to inhibit the MET signaling pathway

Different strategies have been developed to inhibit the MET signaling pathway in NSCLC, including MET TKIs, HGF antagonists (such as rilotumumab [AMG-102] and ficlatuzumab [AV-299]), and anti-MET monoclonal antibodies (such as onartuzumab and emibetuzumab).

MET TKIs are commonly classified into type I, type II, and type III. Type I MET TKIs are ATP-competitive and bind to MET in its active form, subdivided into type Ia and type Ib. Type Ia (crizotinib) is a multikinase inhibitor, and type Ib inhibitors are selective inhibitors, including capmatinib, tepotinib, and savotinib. Crizotinib was initially approved for the treatment of ALK-positive NSCLC and ROS-1-positive NSCLC by the FDA, and it was the first targeted therapy demonstrating a partial response rate in 69 advanced NSCLC patients with METex14 in a phase I PROFILE 1001 study (ORR = 32%, mPFS = 7.3 months) [245]. Subsequently, several phase II studies evaluating the efficiency of crizotinib for the treatment of patients with METex14 have been conducted, including the METROS trial (NCT02499614), the MATCH trial (NCT02465060), and the Matrix trial (NCT02664935). Type Ib inhibitor capmatinib has shown potent efficacy in MET-positive NSCLC, and it was approved for the treatment of metastatic NSCLC patients with METex14 by the FDA on May 6, 2020, based on the results of the phase II GEOMETRY mono-1 trial [246]. Tepotinib has been approved as the first and only once-daily oral MET inhibitor for metastatic NSCLC patients with METex14. It showed promising efficiency and safety in the open-label phase II VISION study (ORR = 46%, DCR = 65.7%) [247]. Savolitinib is a highly selective and potent type Ib MET TKI that effectively blocks HGF-dependent MET phosphorylation and tumor growth [248]. A phase II clinical trial conducted in China demonstrated the efficacy and safety of savolitinib in patients with locally advanced or metastatic METex14-positive NSCLC, especially those with the pulmonary sarcomatoid carcinoma subtype (mOS = 12.5 months, 24-month OS rate = 31.5%, IRC ORR = 49.2%) [249, 250].Type II MET TKIs are also ATP-competitive but bind to MET in its inactive form, such as cabozantinib, merestinib, and glesatinib. Cabozantinib is a multikinase MET inhibitor, and a phase II study (NCT03911193) was conducted to evaluate its efficiency. It was found in a case report that cabozantinib has intracranial penetration and activity and may overcome the acquired resistance and intracranial progression of crizotinib [251]. Tivantinib, a type III MET TKI, has been reported to be non-ATP competitive and highly specific [252]. The efficiency of erlotinib plus tivantinib versus chemotherapy in patients with locally advanced or metastatic NSCLC has been evaluated in a phase II study. The results were not inspiring, in which the mPFS of combined therapy was 7.3 weeks and the PFS of chemotherapy was 18.6 weeks (NCT01395758). The phase III MARQUEE study evaluated the efficiency of erlotinib combined with tivantinib and the combination of placebo with erlotinib. The combination of tivantinib with erlotinib was well tolerated and increased PFS but did not improve OS in nonsquamous NSCLC. The study was terminated due to the failure of the interim analysis [253]. Some novel strategies are also under exploration, such as protein degradation with protein targeting chimeras (PROTACs). PROTACs are multiple MET-targeting inhibitors which can restore the ubiquitination of METex14 [254].

There was a long struggling history of developing drugs against the HGF/MET pathway, particularly with anti-MET/HGF antibodies. Different from MET inhibitors suppressing the kinase domain of MET, antibodies against HGF/MET inhibit the signaling pathway, including neutralizing binding with ligand, preventing receptor dimerization, inducing receptor internalization and degradation, attenuating tumorigenic signaling, and enhancing cell-mediated immune activity [255]. Emibetuzumab is a humanized IgG4 antibody that blocks the binding of HGF to MET and leads to MET degradation. The phase II study evaluated the efficiency of emibetuzumab combined with erlotinib for EGFR-positive patients, and it was found that high MET expression was a negative prognostic marker for patients [256]. Onartuzumab is a humanized single-arm anti-MET antibody blocking the binding of the HGF α-chain to MET, which performed poorly in a phase III study examining the efficiency of erlotinib with or without onartuzumab in patients with MET-positive locally advanced or metastatic NSCLC [257, 258]. Rilotumumab is a fully humanized IgG2 monoclonal antibody that binds to HGF, blocks binding to the c-MET receptor, and blocks downstream activation of the signaling pathway. Rilotumumab was once used with erlotinib in a phase I/II study for advanced NSCLC (NCT01233687), with a DCR of 60% for combination therapy. However, clinical trials of rilotumumab have been terminated based on a planned safety review [259]. Ficlatuzumab is a high affinity and potent anti-HGF IgG1 monoclonal antibody blocking HGF/c-MET binding, but the combination therapy of ficlatuzumab plus gefinitib did not show significant antitumor activity [260].

Although the current efficacy of anti-MET/HGF monoclonal antibodies is not obvious, some new studies have shown promising results of bispecific antibodies such as amivantamab (JNJ61186372) and antibody-drug conjugates (ADC) such as telisotuzumab vedotin (teliso-v, ABBV-399). Amivantamab, a bispecific monoclonal antibody targeting EGFR and MET, received its first FDA approval for the treatment of patients with NSCLC harboring EGFR exon 20 insertion mutations on 21 May 2021 [261]. Given the bispecific nature, amivantamab was observed to have antitumor activity in 9/16 patients with METex14 NSCLC in the CHRYSALIS study [262]. Teliso-v is an ADC comprised of the anti-c-MET monoclonal antibody ABT-700 and cytotoxic microtubule inhibitor monomethyl auristatin E (MMAE). A phase II study (NCT03539536) is ongoing to evaluate the efficiency of teliso-v in patients with previously treated MET-positive NSCLC. In the EGFR WT group, the ORRs in the c-METt high group and in the c-MET intermediate group were 53.8% and 25.0%, respectively [263].

#### Resistance mechanisms to MET-targeted therapies

**Fig. 5:**
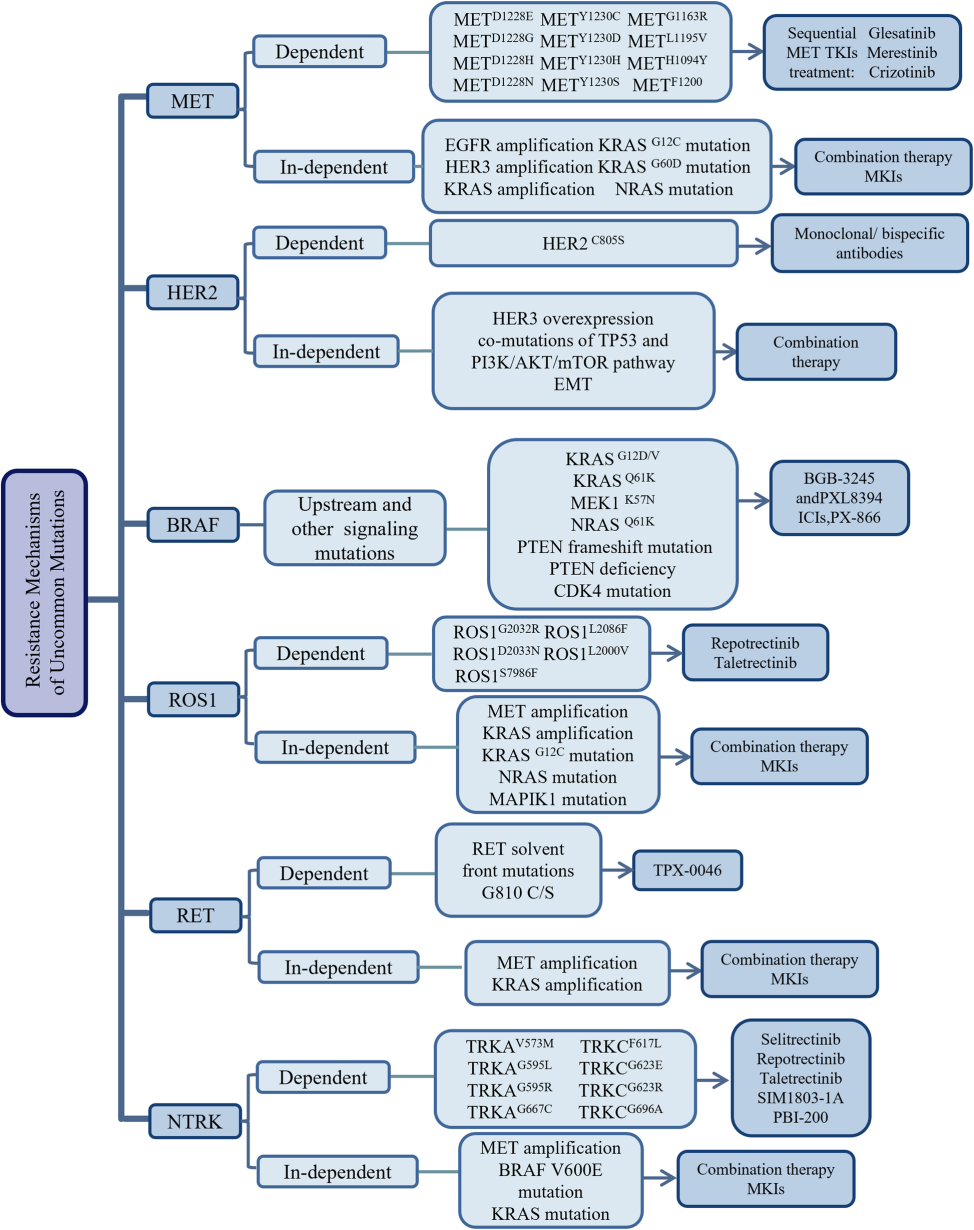
Resistance mechanisms of uncommon mutations. Resistance mechanisms of uncommon mutations of NSCLC (less than 5% cases of NSCLC) are listed in the figure. The first column shows uncommon mutations including MET, HER2, BRAF, ROS1, RET, and NTRK. The second column shows the classification of resistance mechanisms. The third column shows detailed mutations of resistance mechanisms. The fourth column shows current treatment strategies targeted those resistance mechanisms. MET, hepatocyte growth factor receptor; HER2, human epidermal growth factor receptor 2; NTRK, Neurotrophic tyrosine receptor kinase; ROS1, proto-oncogene receptor tyrosine kinase ROS1; BRAF, V-raf murine sarcoma oncogene homolog B1; RET, proto-oncogene tyrosine-protein kinase receptor Ret; EMT, epithelial to mesenchymal transition; MKIs, multikinase inhibitors

While MET-targeted therapies remain immature, the appearance of acquired resistance to MET TKIs has marred the promising efficiency of MET TKIs. Using plasma and tissue NGS to study the genomic alterations occurring at the time of progression on MET TKIs, it was found that on-target mutations accounted for approximately 35%, off-target mutations accounted for 45%, and 25% of resistance mechanisms remained unknown [264]. Focusing on on-target resistance mechanisms, secondary MET kinase domain mutations have been identified in multiple MET TKIs: G1163R, D1228E/G/H/N, Y1230C/D/H/N/S, L1195V mutations (type Ia MET TKI), D1228N (type Ib MET TKI), and H1094Y, L1195V, and F1200 (type II MET TKI) [264, 265]. It seems that each MET TKI has distinct secondary MET mutations in the proportion and mutation spectra, and resistance mutations against type II are sensitive to type I, and vice versa [265]. Amplification of EGFR, HER3, KRAS, and BRAF, as well as activating KRAS mutations, are identified as off-target mutant resistance to MET TKIs [264, 266, 267]. Alterations of other pathways contributing to acquired resistance to MET TKIs were also found in other types of tumors, such as overexpression of PI3K p110α in gastric cancer and upregulation of mTOR, STAT3, and COX-2 in glioblastoma [268, 269]. The resistance mechanisms of MET have been presented in Fig. 5.

#### Combination therapy with MET TKIs

MET amplification can be detected in about 5–22% of NSCLC with acquired drug resistance to first- and second-generation EGFR TKIs [62, 270, 271] and 15–19% of third-generation EGFR TKIs [272, 273]. Besides, in consideration of the correlation of EGFR and the MET pathway, researchers have tried to add EGFR TKIs to MET TKIs in the treatment. Several clinical trials have adopted this regimen to overcome drug resistance. The phase Ib TATTON study assessed the safety, tolerability, pharmacokinetics, and preliminary antitumor activity of savolitinib in combination with osimertinib (NCT02143466). Sequist and his colleagues considered two expansion cohorts: part B and part D. In part B, patients were those who had been or had not been pretreated with a third-generation EGFR TKI who were either Thr790Met positive or negative, and patients in part B received savolitinib 600 mg plus osimertinib 80 mg daily. In part D, patients were those who had not been pretreated with a third-generation EGFR TKI and received savolitinib 300 mg plus osimertinib 80 mg daily. The results of the study demonstrated that the ORR in cohort B was 48% and 64% in cohort D, and adverse effects were less in cohort D, in which patients received lower doses of savotinib [274–276]. The results illustrated the superiority of the combination of savolitinib and osimertinib, and SAVANNAH (NCT03778229) is ongoing to confirm the promising results. Furthermore, anti-EGF antibodies were observed to enhance the antitumor activity of tepotinib and capmatinib by the blockade of EGFR, Akt and Erk1/2 phosphorylation [277].

The role of MET in checkpoint inhibition has not been fully explored. A retrospective study revealed that PD-L1 expression in NSCLC is positively correlated with MET amplification. This proved that MET amplification may drive activation in immune suppression and evasion [278]. It was also found that MET amplification might be associated with TMB, high MET mRNA and protein levels, neoantigen load (NAL), and immune-related gene expression levels, leading to longer PFS in immunotherapy cohorts [279]. However, in another retrospective trial of combination immunotherapy conducted in 147 patients, PD-L1 expression (PD-L1 ≥ 1) was 63%, and the median TMB was lower in METex14-altered lung cancers than in unselected NSCLC. The ORR was 17%, and the mPFS was 1.9 months in the 24 response-evaluable patients. Responses were not enriched in high TMB and tumors with PD-L1 expression ≥ 50% [280]. The results indicated that immunotherapy had poor activity in MET alterations, and more investigation is needed to support the potential association between immunotherapy and MET-positive NSCLC patients.

### Oncogenic HER2 alterations

Current studies suggest that alterations in HER2 are an important oncogenic change in NSCLC, presenting in 3% of NSCLC [281]. HER2 belongs to the ERBB family, it lacks a ligand-binding structure and leads to autophosphorylation by dimerization with other ligand-binding receptors in the ERBB family. Autophosphorylation results in activating downstream signaling pathways, leading to uncontrolled cell proliferation and ultimately causing tumorigenesis [282]. Mechanisms of HER2 activation include gene mutation, gene amplification, and protein overexpression. Exon 20 insertion mutation is the most common HER2 mutation. Although the clinical features and prognosis of these three alterations are not identical, her2 mutations are more common in women, never smokers, and patients with lung adenocarcinoma [283].

There are a variety of approaches to target HER2 molecular alterations, including small molecule TKIs, anti-HER2 antibodies, and antibody-drug conjugates (ADCs). Non-selective TKIs such as afatinib and dacomitinib can inhibit the phosphorylation of protein tyrosine residues, thereby blocking downstream signaling pathways. Current clinical studies have shown that non-selective TKIs have limited efficacy in NSCLC patients with HER2 mutation. The ORR of afatinib in HER2-mutated NSCLC did not exceed 20% [284]. The PFS and OS of dacomitinib were 3 months and 9 months, respectively, in HER2 mutant lung cancer [285]. It is interesting to note that afatinib showed clinical benefit in patients with specific mutation subtypes (p.A775_G776inSYVMA), with an ORR of 33%. Patients with such mutations may benefit from targeted treatment with afatinib [286]. Humanized monoclonal anti-HER2 antibodies play a role in antitumor therapy by binding to the HER2 extracellular domain and inhibiting dimerization [287]. Trastuzumab and pertuzumab work very well in breast cancer harboring HER2 alteration, but they demonstrated poor curative effects in NSCLC patients with HER2 alteration [288].

Recently, novel HER2 TKIs have shown good antitumor effects and are being actively studied. Phase II studies for poziotinib (novel selective HER2 TKIs) have appeared to improve outcomes in previously treated NSCLC patients with HER2 exon 20 insertion. The ORR was 27.8%, and mPFS was 5.5 months [289]. Another new generation TKI pyrotinib also exhibited promising efficacy, and the ORR was 30% [290]. In pre-clinical models, mobocertinib has demonstrated antitumor activity in HER2 exon 20 insertion mutants [291]. In addition, ADCs therapy has also achieved good therapeutic results, providing an option for NSCLC patients with HER2 alterations. Trastuzumab–emtansine (T-DM1) is a kind of ADCs that links trastuzumab with a cytotoxic microtubule inhibitor. The combination of monoclonal antibody drugs and cytotoxic drugs can improve efficacy and reduce toxic and side effects [292]. A phase II trial of T-DM1 demonstrated an ORR of 44% and a median PFS of 5 months in HER2-mutated NSCLC [293]. However, another study showed that patients with HER2 amplification did not show good results with TDM-1 [294]. The results have been mixed and more clinical trials about TDM-1 are ongoing. Notably, trastuzumab-deruxtecan (T-DXd, DS-8201), another ADC agent consisting of an anti-HER2 antibody and a topoisomerase I inhibitor, demonstrated promising outcomes in HER2 mutant NSCLC in a phase II study [295]. The DESTINY-Lung 01 trial has shown that the ORR was 61.9% and mPFS was 14 months in NSCLC patients with HER2 mutation [296]. Given the excellent therapeutic effect, T-DXd has been approved by FDA as the only targeted drug in HER2 mutant NSCLC. Patients with HER2 overexpression had less effective treatment than those with HER2 mutations, the ORR was 24.5% and the median PFS was 5.4 months [296]. These agents bring hope to the treatment of HER2-altered NSCLC.

Generally, the resistance mechanisms of HER2 mutations were divided into secondary mutations-mediated and bypass pathway-mediated. Meanwhile, some clinical studies have mentioned that co-mutations of TP53 and PI3K/AKT/mTOR pathway are associated with primary resistance to HER2-targeted therapy in lung adenocarcinomas [297]. Tarloxotinib is a novel pan-HER inhibitor, which is activated in tumor cells to form potent drugs Tarloxotinib-E. A vitro study has linked the secondary C805S HER2 mutation and HER3 overexpression with acquired resistance to Tarloxotinib-E [298]. Therefore, anti-HER3 monoclonal antibodies or bispecific HER2/HER3 antibodies may be potential therapeutic approaches against tarloxotinib-E resistance with HER3 activation [299]. In another in vitro study, secondary C805S was identified as a potential mechanism of acquired resistance to poziotinib. Heat shock protein (HSP) 90 inhibitors may be able to overcome the C805S mutant resistance [300]. In the lung tumor model, T-DXd was shown to be effective against the resistance model of T-DM1. The combination of irreversible pan-HER inhibitors and T-DXd can increase antitumor efficacy by enhancing receptor ubiquitination and ADC internalization [301]. In addition, like other types of resistances in EGFR-mutant NSCLC, EMT may also be possible [49]. To further improve the survival of NSCLC patients with HER-2 alteration, many novel drugs targeting HER-2 or overcoming resistance are under investigation. It is believed that HER-2 positive patients will have more choices in the near future. The resistance mechanisms of HER2 have been presented in Fig. 5.

### Oncogenic BRAF mutations

BRAF is a RAF (rapidly accelerated fibrosarcoma) kinase that is downstream of RAS and signals via the MAPK pathway first detected in 1988 [302]. BRAF mutations are found in 1.5 ~ 8% of NSCLC cases, with a balanced repartition between V600 and non-V600 mutation types, and BRAF V600E mutations seem to be more frequent in female and never-smoker patients [303–305]. BRAF mutant alleles are classified into three subtypes. Class I BRAF mutations (BRAF V600 mutations) are RAS-independent. Class II BRAF mutants (p.G464E/V/R, p.G469A/V/S, and so on) are RAS-independent, resistant to vemurafenib, and sensitive to novel RAF dimer inhibitors or MEK inhibitors. Class III mutants are RAS-dependent and sensitive to ERK-mediated feedback, which may benefit from EGFR-targeted therapy [306–308]. The FDA granted approvals to dabrafenib and trametinib combination for patients with metastatic NSCLC with BRAF V600E mutation on June 22, 2017.

Although two inhibitors were adopted in this treatment regimen, resistance inevitably occurs. Mechanisms of resistance to dabrafenib and dabrafenib-trametinib combination in clinical specimens include mutations in the signaling components directly upstream of BRAF, such as KRAS, leading to the activation of the PI3K/AKT signaling pathway, such as KRAS G12D/V and KRAS Q61K. Other mutations reported from cases include MEK1 K57N, NRAS Q61K, and PTEN frameshift mutations [309–312]. Although the mechanisms of resistance to dabrafenib or dabrafenib-trametinib combination therapy for NSCLC have not been thoroughly described, the activation of the PI3K/AKT/mTOR pathway by PTEN deficiency is considered to be another mechanism. The upregulation of cyclin-dependent kinase 4 (CDK4) was also reported to mediate acquired resistance to dabrafenib combined trametinib in lung cancer patients with BRAF V600E mutation [313].

Other BRAF inhibitors, such as lifirafenib (BGB-283), BGB-3245, and binimetinib (MEK162), have been considered as potential target therapy options for NSCLC with BRAF mutations. However, few of them showed better results compared with current therapy. Like lifirafenib, a reversible inhibitor of BRAF V600E, wild-type A-RAF, B-RAF, C-RAF, and EGFR have antitumor activity and an acceptable risk–benefit profile and in solid tumors patients with BRAF V600-mutation [314]. A phase I study demonstrated that a patient with BARF-mutated NSCLC had unconfirmed PR (NCT02610361) [314].

BGB-3245, a second-generation B-RAF inhibitor, has shown to inhibit tumor cell lines harboring non-V600 B-RAF mutations and was also active toward B-RAF/MAP-ERK kinase (MEK) inhibitor-resistant tumors [315]. A phase I trial to evaluate the effects of BGB-3245 is being conducted in patients with BRAF mutations (NCT04249843). New BRAF inhibitors under investigation, such as PXL8394, have been proven to evade the MAPK pathway and are active in both BRAF V600E and non-V600E mutated patients [316]. Moreover, a study demonstrated that PX-866, an irreversible small molecule panisoform inhibitor of PI3K, can be safely used in combination with a modified dose of vemurafenib in the treatment of patients with advanced BRAF V600-mutated cancers [317]. Above targeted therapy, Dudnik E et al. reported that ICIs have favorable activity both in BRAF V600E- and BRAF non-V600E-mutant NSCLC patients, as BRAF-mutated patients have high levels of PD-L1 expression, low/intermediate TMB and microsatellite-stable status [318].

Although targeted therapy has achieved significant efficacy, resistance is still inevitable in most patients. At present, the mechanism of BRAF-targeted drug resistance in NSCLC patients is very limited and has not been fully elucidated. The resistance mechanisms of BRAF have been presented in Fig. 5.

### ROS1 gene rearrangements

ROS1, encoding a receptor tyrosine kinase (RTK) without a known ligand, is found on chromosome 6q22 and shows a structure similar to that of the ALK gene. ROS1 fusions occur in 1% to 2% of NSCLC patients and are often associated with younger age, generally in light or nonsmokers [319]. All ROS1 gene fusions harbor the ROS1 kinase domain, among them, CD74-ROS1 is the most frequent variant (38–63%), followed by EZR (13%), SDC4 (13%), and SLC34A2 (10%) [320, 321]. ROS1 rearrangements do not overlap with mutations in other oncogenic drivers, such as EGFR or ALK. Crizotinib and entrectinib were approved by the FDA for the treatment of NSCLC patients with ROS1 rearrangement in 2016 and 2019, respectively [322, 323]. Furthermore, ceritinib also demonstrated potent clinical activity in previously treated ROS1-rearranged NSCLC patients [324]. The NCCN guideline recommends crizotinib, entrectinib, and ceritinib as first-line treatments for ROS1-positive NSCLC patients. In addition, lorlatinib is listed as a subsequent option when ROS1-positive NSCLC patients progressed on crizotinib or entrectinib [325].

Crizotinib and entrectinib showed promising antitumor efficacy in ROS-positive patients. Sadly, resistance to these two TKIs is a frequent occurrence. The median PFS of patients using crizotinib was 19.2 months [326]. The median PFS of entrectinib was 19.0 months, although it is highly penetrated and remains in the CNS [327]. The most common ROS1 resistance mutation caused by first-generation TKIs was ROS1 G2032R, which was detected in approximately one-third of cases. Other on-target ROS1 resistance mutations include D2033N, L2026M, S1986F/Y, L2086F, L2000V, G2032K, and so on. Among them, G2032, D2033, and L1951 are substitutions at solvent-front residues, and L2026 is a ‘gatekeeper’ residue [328–330]. Some specimens lacking on-target mutations had other mutations, such as MET amplification, KRAS amplification, KRAS G12C mutation, NRAS G60E mutation, MAP2K1 mutation, in-frame deletions in MEK1 (MEK1delE41_L54), MEKK1, NF1 loss-of-function mutations, BRAF V600E mutation, PIK3CA E545K mutation and so on [321, 331, 332]. In preclinical models, the acquisition of KRAS G12C mutation and the amplification of KRAS and FGF3 was reported to mediate the resistance to entrectinib [333]. The resistance mechanisms involved in TKIs targeting ROS1 rearrangement vary, and more studies are necessary to comprehensively assess genetic mediators of resistance to ceritinib and entretinib.

To overcome the resistance mechanism, next-generation TKIs are under investigation in preclinical and clinical settings. Repotrectinib and taletrectinib are effective targeted therapies against ROS1-positive NSCLC patients who are TKI-naïve or TKI-resistant [334]. Repotrectinib (TPX-0005), a new generation ROS1/ALK/TRK inhibitor that can overcome the G2032R resistance mutation, is under evaluation in clinical practice in the TRIDENT-1 study (NCT03093116). The positive outcomes in patients who developed the G2032R resistance mutation after crizotinib may suggest a sequential strategy with ROS1 inhibitors. Another TKI, taletrectinib (DS-6051b/AB-106), is an inhibitor of ROS1 and NTRK with potent preclinical activity against the ROS1 G2032R solvent-front mutation, among others. Preliminary efficacy was observed in patients with crizotinib-refractory ROS1-positive NSCLC [334–336]. Of the novel TKIs reported to overcome on-target resistance, several other TKIs have also shown antitumor efficacy in patients who are refractory to previous targeted therapy. Cabozantinib is especially effective against solvent-front ROS1 resistance mutations, such as ROS1 L2086F, ROS1G2032R/L2086F, and ROS1S1986F/G2032R/L2086F in Ba/F3 models [331] and D2033N in clinical settings [337]. A phase II trial to evaluate the effects of cabozantinib is being conducted in patients with ROS1 rearrangement. (NCT01639508) Besides, brigatinib inhibits the phosphorylation of ROS1 and ERK in CD74-ROS1 wild-type and L2026M mutant-transformed Ba/F3 cells [338]. Several clinical trials evaluating the activity of brigatinib in ROS1-positive NSCLC patients are recruiting (NCT03868423, NCT04005144, NCT04591431). Above targeted therapy to overcome on-target resistance, Zhou and colleagues reported that a ROS1-positive patient with the G2032K mutation after lorlatinib treatment responds to nab-paclitaxel plus pembrolizumab, indicating that the combination of ICIs and chemotherapy may be considered as a treatment option in those patients [339].

Additionally, resistance driven by bypass mechanisms can be treated by combining ROS1 inhibitors with other inhibitors or by adopting targeted therapy for subsequent mutations [340]. For example, dabrafenib and trametinib combination therapy showed potential efficacy and may serve as an effective option for later-line treatment for patients harboring resistant BRAF V600E after ROS1 TKI treatment [341]. For those patients who harbor off-target resistance but without a known targeted therapy, chemotherapy and ICIs may provide a treatment option. A retrospective study reported that patients with ROS1 fusion may benefit from pemetrexed-based chemotherapy [342]. The combination of targeted therapies with ICIs or chemotherapy has been thought to be an option for multidrug-resistant patients [342], and a series of clinical trials (NCT04989322, NCT04042558) are under investigation to determine the efficacy of combination therapy. The resistance mechanisms of ROS1 have been presented in Fig. 5.

Currently, there are three agents approved in the first-line treatment of ROS1-positive NSCLC, although resistance to available ROS1 inhibitors presents a significant clinical challenge. Except for the absence of on-target and off-target mutations listed, there were other cases showing resistance without any positive results, which demands an in-depth characterization of resistance.

### Oncogenic RET mutations

The RET receptor tyrosine kinase is oncogenically activated by RET gene fusions in 1% to 2% of NSCLC. The RET gene encodes a receptor tyrosine kinase that signals involved in normal embryonic development [343, 344]. Currently, more than ten fusion partners have been identified, among them, the main partner of RET rearrangement is the KIF5B protein, which accounts for 62% of all RET gene rearrangement variants [345]. Those patients with RET fusion seemed to share common clinicopathological characteristics, including younger age, never-smoker status, early brain metastases, and poor differentiation [346–348]. The TKIs selpercatinib and pralsetinib were approved by the FDA for the treatment of advanced RET fusion-positive lung cancer in 2020. The approval was based on the encouraging results of the LIBRETTO-001 and ARROW Phase 1/2 trials, respectively, in which both selpercatinib and pralsetinib demonstrated robust efficacy. The ORRs among patients previously received platinum-based chemotherapy are 55–64% and 66–85% among treatment-naïve patients with RET fusion-positive NSCLC [349, 350].

Although the ratio of resistant cases without RET resistance mutations is strikingly low, resistance is still a major challenge in RET fusion-positive lung cancer treated with RET TKIs. Generally, the resistance mechanisms of RET TKIs are classified as RET-dependent and RET-independent resistance. Some studies identified RET solvent front mutations G810 C/S as recurrent mechanisms of resistance [351]. Other acquired oncogenic mutations are RET-independent resistance, including amplification of MET and KRAS [351]. The resistance mechanisms of RET have been presented in Fig. 5.

To overcome the resistance mechanisms of RET TKIs, next-generation inhibitors are under investigation. TPX-0046, a potent RET/SRC inhibitor, showed antitumor potency against RET G810 solvent front mutation cancer models [352]. Currently, a phase I clinical trial recruiting patients with advanced RET-altered solid tumors is being carried out to evaluate this novel TKI (NCT04161391).

Some studies have suggested that combination strategies might be a treatment option for patients with RET-independent resistance. Both the combination of two TKIs and the application of multikinase inhibitors (MKIs) are promising approaches. Rosen et al. reported that selpercatinib plus crizotinib demonstrated clinical activity in NSCLC patients with concurrent RET fusions and MET amplification, revealing a possible treatment strategy. Furthermore, cabozantinib, an MKI targeting several molecular pathways, may represent an alternative option for patients who develop MET amplification after TKI treatment.

However, due to the low incidence of RET rearrangement in NSCLC and limited exploration of resistance mechanisms, further preclinical and clinical studies are needed to clarify the resistance mechanisms and effectively overcome resistance in these patients.

### Oncogenic NTRK mutations

NTRK including NTRK1, NTRK2, and NTRK3, encodes the neurotrophin receptors TRKA, TRKB, and TRKC, respectively. The NTRK signaling pathway plays an important role in the development of the central and peripheral nervous systems by being involved in cell proliferation, differentiation, and apoptosis [353]. NTRK fusion is rarely detected in lung adenocarcinoma (less than 1% of cases), and the commonly reported NTRK fusions include TPM3-NTRK1, MPRIP-NTRK1, CD74-NTRK1, and ETV6-NTRK3 fusions [354, 355]. Fusions involving TRK protein tyrosine kinases are not only oncogenic drivers in lung cancer. Xia and colleagues also reported that the emergence of NTRK1 fusion might act as a resistance mechanism to EGFR TKIs [356]. Larotrectinib, a first-generation NTRK TKI, effectively inhibits the growth of NTRK fusions positive cell lines or xenografts and is associated with inhibition of the downstream RAF-MEK-ERK or PI3K-AKT pathways [357]. It is also highly active in TRK fusion-positive solid tumor patients, with an ORR of 79%, and 16% of patients had CR [358]. Similarly, entrectinib inhibits the growth of cell lines or xenografts containing LMNA-NTRK1 or EVT6-NTRK3 mutations, as well as the downstream pathway signaling [359], and entrectinib also induced durable responses in patients with NTRK fusion-positive solid tumors [360]. Based on the positive results of these two first-generation TRK inhibitors, the NCCN guideline recommends larotrectinib and entrectinib as treatment for NTRK fusion NSCLC patients [361].

Although these TKIs showed potential antitumor efficacy in NTRK fusion-positive patients, on-target and off-target resistance mechanisms eventually developed to NTRK inhibitors. On the one hand, several point mutations were identified that confer resistance to larotrectinib and entrectinib, including TRKA V573M, G595L, G595R, G667C, TRKC F617L, G623E, G623R, and G696A. In particular, the mutations on TRKA F589 and TRKC F617 were paralogs to ALK L1196M, ROS1 L2026M, and EGFR T790M, which were identified in other patients who have progressed on TKI therapy.

These mutations involve three major conserved regions: the solvent front, the gatekeeper residue, and the xDFG motif [362, 363]. These amino acid substitutions may result in structural changes and even affect TRK inhibitor binding [364]. On the other hand, similar to ALK and ROS1 fusion-positive lung cancers, patients with NTRK fusion also can develop off-target resistance to TKI therapy, mechanisms including MET amplification, BRAF V600E mutation, or hotspot mutations involving KRAS [365]. As a number of patients developed resistance to first-generation TKIs, novel treatment strategies are emerging to overcome on-target and off-target resistance mechanisms. The resistance mechanisms of NTRK have been presented in Fig. 5.

Selitrectinib (LOXO-195) is a promising selective TRK TKI which was designed to treat patients progressed on first-generation TRK TKIs. The activity of selitrectinib against above mentioned acquired mutations was confirmed in both in vitro and in vivo experiments [366]. Selitrectinib is currently in phase I/II clinical trials for the treatment of patients with NTRK fusions (NCT03215511), and preliminary results showed that patients who failed on larotrectinib and developed solvent front substitution-mediated acquired resistance also responded well to selitrectinib [366]. In total, 31 cancer patients with TRK fusion- received selitrectinib; however, all 3 patients with identified bypass resistance mechanisms did not respond to selitrectinib. In addition, none of the 5 patients with unknown resistance mechanisms responded to therapy [366]. These results indicated that other approaches are needed to conquer the off-target resistance mechanism. Another next-generation TRK inhibitor, repotrectinib (TPX-0005), was rationally designed to overcome the resistance mediated by solvent-front and gatekeeper mutations. Compared with selitrectinib, it was more potent against all tested resistance mutations. Repotrectinib caused tumor regression in preclinical xenograft models harboring resistance mutations [362]. In clinical settings, it also brings durable responses in both TKI-naïve and TKI-pretreated patients (NCT03093116) [363]. Taletrectinib (also known as AB-106 or DS-6051b) showed preliminary antitumor efficacy in patients with NTRK fusions but with a very limited number of patients.

Above selitrectinib and repotrectinib, other selective NTRK inhibitors are also currently being evaluated in early phase I/II trials, such as PBI-200 (NCT04901806), SIM1803-1A (NCT04671849), and taletrectinib (NCT04617054). Multikinase inhibitors are under investigation to treat patients with off-target resistance mechanisms. The application of merestinib (NCT02920996) and cabozantinib (NCT01639508) in advanced solid tumors with actionable genetic mutations are under clinical investigation. Combination therapy has been shown to reestablish disease control in off-target resistance cases. In a case of fusion-positive cancer patients with MET amplification-driven resistance to a first-generation TRK inhibitor, the patients achieved CR with the combination therapy of a TRK and MET inhibitor [365]. Reestablishing disease control indicated the importance of rebiopsy, genomic detection after progression, and the blockade of a concurrently activated receptor tyrosine kinase.

### Other rare mutations and pathways of NSCLC

There are some other rare mutations in NSCLC patients, such as HER2 exon 20 in-frame insertions/amplification, FGFR1 amplification, FGFR2–4 mutation, NGR1 gene fusion/translocation/mutation, and the PI3K pathway, including pTEN protein loss, PI3K amplification and mutation, and pTEN R233* and AKT1 mutation. The incidence of these mutations is strikingly low, and the specific TKIs targeting these pathways are still under investigation. The mechanisms of resistance are less clear and need further study.

## Conclusions and future perspective

In the last tens of decades, targeted therapies have changed the treatment landscape of NSCLC to a great extent since EGFR TKIs are applied in the clinic. These great advances are largely attributed to NGS technologies applied in routine molecular pathology and the wide application of liquid biopsy in initial and resistance mechanism molecular analysis. The target driver genes of drugs approved by the FDA include EGFR mutations (L858R, 19 del, T790M), KRAS mutations, ALK rearrangements, MET alterations, and so on. Those target driver gene mutations identified demonstrated great superiority in survival outcomes including PFS, OS, and ORR compared with platinum-based chemotherapy. In addition, new driver gene mutations have been continuously identified, and molecular targets such as microRNAs and HER3 have been reported. TKIs targeting these mutations were approved in succession, and numerous compounds have been explored in clinical trials. However, challenges remain in introducing these investigational molecular targets into clinical practice, including the issue of resistance to targeted agents and the appropriate sequence of drug administration. Multiple studies have shown that targeted drugs will eventually develop resistance, and the resistance mechanisms include 1) primary resistance, 2) dependent resistance, 3) activation of bypass or downstream pathways, and 4) histological transformation. Although the development of targeted drugs is underway at a furious pace, the current rate of drug development has not been able to chase the findings of resistance mutations. In addition, with the development of drugs, the sequence of administration of a variety of targeted drugs also needs great attention. Furthermore, combination strategies such as targeted therapy and immunotherapy have been explored in numerous clinical settings. In the complex mutational environment of NSCLC, inhibition of multiple pathways to control cancer seems to be a more effective treatment option. However, the survival rate of many combination therapies remains unknown. A deeper understanding of the various pathways of NSCLC tumorigenesis and growth experience, individualized assessment at baseline, to tailor treatment strategies for patients, and continuous assessment in treatment is an important means at present. The promise of targeted therapy for NSCLC will continue to evolve in the coming years. With these advances and future work, the survival of NSCLC patients will be further improved, and our understanding of lung cancer will be more comprehensive.

## Supplementary Information

**Additional file 1.**

## Data Availability

Not applicable.
